# From Evasion to Collapse: The Kinetic Cascade of TDP-43 and the Failure of Proteostasis

**DOI:** 10.3390/ijms27031136

**Published:** 2026-01-23

**Authors:** Angelo Jamerlan, John Hulme

**Affiliations:** Department of Bionanotechnology, Bionano Research Institute, Gachon University, Seongnam-si 13120, Gyeonggi-do, Republic of Korea; angelo@gachon.ac.kr

**Keywords:** TDP-43 proteinopathy, proteostasis collapse, ubiquitin-proteasome system (UPS), autophagy-lysosome pathway (ALP), phase separation, post-translational modifications (PTMs), amyotrophic lateral sclerosis (ALS), frontotemporal dementia (FTD), neurodegeneration

## Abstract

Amyotrophic lateral sclerosis (ALS) and frontotemporal dementia (FTD) are devastating neurodegenerative diseases that, despite the availability of symptomatic and modestly beneficial treatments, still lack therapies capable of halting disease progression. A histopathological hallmark of both diseases is the cytoplasmic deposition of TDP-43 in neurons, which is attributed to both intrinsic (e.g., mutations, aberrant cleavage) and extrinsic factors (e.g., prolonged oxidative stress, impaired clearance pathways). Mutations and certain PTMs (e.g., cysteine oxidation) destabilize RNA binding, promoting monomer misfolding and increasing its half-life. Disruptions to core ubiquitin-proteasome system (UPS) subunits impede efficient processing, contributing to the clearance failure of misfolded TDP-43 monomers. The accumulation of monomers drives phase separation within stress granules, creating nucleation hotspots that eventually bypass the thermodynamic barrier, resulting in exponential growth. This rapid growth then culminates in the failure of the autophagy-lysosome pathway (ALP) to contain the aggregation, resulting in a self-sustaining feed-forward loop. Here, we organize these factors into a conceptual kinetic cascade that links TDP-43 misfolding, phase separation, and clearance failure. Therapeutic strategies must therefore move beyond simple clearance and focus on targeting these kinetic inflection points (e.g., oligomer seeding, PTM modulation).

## 1. Introduction

Trans-active response DNA-binding protein (TDP-43) is a versatile RNA-binding protein that plays roles in RNA splicing, RNA stabilization, mRNA transport, gene expression control, and the regulation of mRNA metabolism within stress granules, which are sites of translational repression under stress conditions [1,2,3]. It is also critical for embryonic and neural development; for instance, TDP-43 knockout models of mice and zebrafish exhibited embryonic lethality and motor deficits [4]. Alternative splicing, maintaining RNA stability, and processing non-coding RNAs in transcripts are all critical for cell differentiation and organogenesis. The loss of TDP-43 disrupts these processes, resulting in failed development [4] and preventing the expression of synaptic proteins necessary for neurodevelopment, which leads to motor deficits [4].

TDP-43 is primarily localized in the nucleus, and a fraction of its total cellular concentration can be found in the cytoplasm, where it performs its other functions. Its subcellular distribution was thought to be regulated by two elements: a nuclear localization signal (NLS) and a nuclear export signal (NES). The NLS (residues 82–98) tags TDP-43 for import into the nucleus, ensuring that the protein stays there in a soluble state [5,6]. Conversely, the NES (residues 239–250), initially thought to be required for protein egress, was later shown to be non-functional [5,7]. Nevertheless, TDP-43 has been shown to have additional mechanisms, such as the self-regulation of its own mRNA, which allows it to adjust its expression when it becomes overabundant in other cellular regions [8].

Despite this, mutations, prolonged cellular stress, and aberrant post-translational modifications (PTMs) can cause the protein to misfold and form irreversible aggregates that accumulate in the cytoplasm [9,10]. These cytoplasmic aggregates are a hallmark observation in TDP-43 proteinopathies, namely amyotrophic lateral sclerosis (ALS) and frontotemporal dementia (FTD) [11,12,13]. Recent neuropathological findings of TDP-43 inclusions concentrated in the hippocampus and limbic structures have also been designated as limbic-predominant age-related TDP-43 encephalopathy (LATE), a condition that becomes more common in older populations (present in more than 20% and up to roughly half of individuals over 80 years in community-based autopsy cohorts) and appears to mimic Alzheimer’s disease (AD) but is primarily driven by TDP-43 proteinopathy [14]. Here, essentially the same kinetic cascade of TDP-43 misfolding, aggregation, and clearance failure appears to apply, but in a cellular milieu that is heavily influenced by advanced aging and its cumulative stresses [14]. In these diseases, the formed TDP-43 oligomers further accelerate the pathological process, acting as seeds and sequestering free and functional TDP-43, eventually leading to nuclear depletion [15,16]. This leads to devastating neurodegenerative consequences, as the protein can no longer perform its regulatory functions required for gene expression and alternative splicing, which are essential for neuronal maintenance and survival.

Nevertheless, several compensatory pathways within the cell help identify and process disordered biomolecules for degradation and clearance. For disordered proteins (e.g., pathologic TDP-43), these checkpoints are in the form of molecular chaperones, addition of PTMs, small ubiquitin-like modifier (SUMO). SUMO-Ubiquitin networks, packaging in lysosomes, aggresomes, and exosomes [9,17,18,19,20]. However, these aggregates possess intrinsic properties and external factors within their immediate cellular environment that enable them to evade these checkpoints and remain in the cytoplasm. In this review, the functional attributes of pathological TDP-43 and their disruptive roles in TDP-43 proteostasis, sequestration, and catabolic clearance will be discussed. Elucidating the mechanistic details of how pathological TDP-43 circumvents these pathways will aid in understanding the pathogenesis of similar proteinopathies and inform the development of actionable targets for therapeutic applications and biomarker discovery.

## 2. TDP-43 Is Intrinsically Aggregation-Prone Due to Its C-Terminal Domain

### 2.1. Structural Domains and Aggregation-Prone Regions

**Figure 1:**
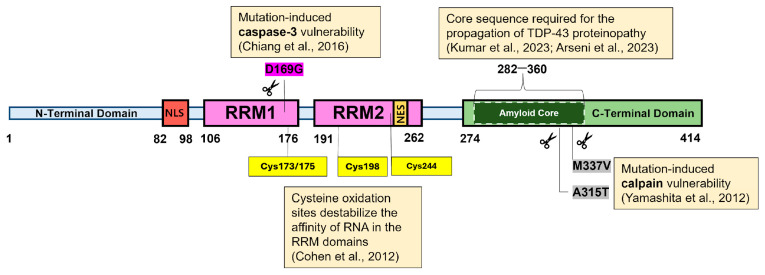
Structural overview and pathogenic hotspots of TDP-43 discussed in this review. The full-length protein (414 amino acids) comprises the N-terminal domain (NTD), two RNA recognition motifs (RRM1 and RRM2), and the highly disordered C-terminal domain (CTD). The NLS, which is responsible for maintaining nuclear localization of the protein, is shown near the NTD. The NLS is within the RRM2 and was shown to be nonfunctional. The amyloid core within the CTD is an essential sequence required for aggregate formation. Mutations such as D169G, M337V, and A315T indicate sites that become more vulnerable to cleavage by either caspase-3 or calpains. Cysteine oxidation sites that destabilize the interaction between RNA and the RRM domains are also shown [22,29,30,31,32].

TDP-43 is a highly conserved protein made up of 414 amino acids. Its full length is organized into four major domains: (1) an N-terminal domain (NTD), that spans approximately 1–80 residues; (2) two RNA Recognition Motifs (RRMs, at residues 105–169 and 194–257, respectively), which are a characteristic feature of heterogeneous nuclear ribonucleoprotein (hnRNP) proteins responsible for RNA recognition and protein interactions; and (3) a highly disordered glycine-rich C-terminal domain (CTD) or prion-like domain (residues 277–414) (Figure 1) [21,22]. Other studies refer to the CTD as the glycine-rich region (GRR) or the low-complexity domain (LCD). LCDs in RNA-binding proteins (RBPs) are typically enriched in a small subset of amino acids (e.g., G, Q/N, S, Y, F, R), which reduces sequence diversity and results in “low sequence complexity.” The absence of side-chains in glycine confers flexibility, making these domains key regulators of protein phase behavior, especially in scenarios involving regulation and stress adaptation [23].

The CTD is unstructured and contains most of the documented ALS-associated mutations, thus it is considered a significant driving force of protein assembly [24,25]. Studies have revealed that the liquid droplet environment, electrostatic repulsion, the presence of CTD segments that act as steric zippers, and low-complexity aromatic-rich kinked segments (LARKS) that can be converted to irreversible interactions by fALS variants are all located in the CTD and play a common role in increasing the rate of TDP-43 aggregation [26,27]. As for the other TDP-43 domains, molecular modeling revealed different segments from the NTD (^24^GTVLLSTV^31^), RRM1 (^128^GEVLMVQV^135^), and RRM2 (^247^DLIIKGIS^254^) as aggregation-prone and were confirmed to have amyloid-fibril propensities through various biophysical techniques and molecular dynamics simulations [28]. The simulations revealed that the RRM2 peptides transitioned from random coil to α-sheet-rich structures, forming parallel and anti-parallel α-sheets, even in the absence of aromatic residues, underscoring RRM2 as a critical structural region for TDP-43 aggregation [28].

Complementing these earlier findings on aggregation-prone regions, Kumar et al. applied proteolytic cleavage to recombinant TDP-43 filaments, revealing a hidden core sequence within the CTD (residues 279–360), which was found to be necessary for the propagation of TDP-43 proteinopathy [22]. Notably, this core sequence resembled that described by Arseni et al. when they isolated TDP-43 pathological filaments from the brains of ALS/FTLD-TDP (type A) patients, a subtype of FTLD (the other subtype is FTLD-tau) [29]. This core sequence (residues 282–360) had a double-spiral shape fold, whereas the former (in vitro) was buried and flanked by the NTD and RRMs (Figure 1) [22,29]. The findings suggest that the cellular environment and flanking sequences are important contributors to the final filament orientation. These differences likely originate from non-proteinaceous cofactors found in filament cavities and disease-specific PTMs (e.g., citrullination and monomethylation of R293), which have been shown to promote specific amyloid folds and stabilize structural variations in the human brain [22,29]. The emergence of these differences in fold structure, despite primary sequence similarities, underscores the importance of assessing full-length proteins in their native contexts.

### 2.2. Intrinsic Self-Regulatory Mechanisms

Although the CTD of TDP-43 is intrinsically disordered and predisposed to aggregation, Afroz et al. (2017) [33] showed that the NTD also interacts to form physiological oligomers under stress. The formed TDP-43 oligomers result in the spatial separation of the CTD ends, minimizing the chances of contact between these regions and thereby reducing aberrant aggregation [33]. This suggests that TDP-43 has evolved an intrinsic self-regulatory mechanism to counteract its own tendency to aggregate. These findings were supported in a subsequent paper by Jiang et al. (2017), which showed that the NTD is primarily responsible for the formation of homodimers in solution, and further tetramerization occurs through intermolecular disulfide bonds [34]. More importantly, they showed that mutations destabilizing the NTD dimer significantly enhanced the formation of cytoplasmic inclusions, indicating that NTD dimerization is essential in protecting against aggregation [34].

Another study by Wang et al. (2018) [35] further demonstrated the critical role of NTD in enhancing TDP-43 polymeric assembly and liquid-liquid phase separation (LLPS), where proteins and RNA condense into dynamic, droplet-like compartments. Since the interaction of NTD is much weaker and less spontaneous than CTD, the authors hypothesized that TDP-43 phase separation is favored through the synergistic cooperation of NTD and CTD interactions [35]. Solubility shifts in TDP-43 constructs made of NTD alone, CTD alone, and full-length TDP-43 were compared using turbidity assays and microscopic observation of droplet formations. The LLPS of full-length TDP-43 was found to be more pronounced than that of CTD alone, suggesting the contributory role of NTD interaction sites [35]. As for NTD alone, the construct does not readily undergo LLPS but instead forms weak, reversible head-to-tail polymers in a linear arrangement. Most importantly, introducing a phosphomimetic substitution (S48E) in the NTD disrupted head-to-tail assembly, thereby weakening and raising the concentration threshold for LLPS, despite the CTD’s inherent ability to phase separate, further validating the synergistic involvement of the NTD in phase separation [35]. These findings, together with more recent work showing that RNA binding and macromolecular assembly regulate TDP-43 LLPS behavior in vivo, support the idea that phase separation is a tightly controlled, sequence-encoded property, rather than an inevitable prelude to aggregation [35,36,37]. Recent studies also showed that intact RNA binding can buffer TDP-43 against aberrant LLPS and aggregation, whereas RNA-binding-deficient or acetylation-mimicking mutants undergo distinct, aggregation-prone phase behavior [16,38].

While the cooperation between the NTD and the CTD underscores how TDP-43 regulates the functional assembly of oligomers with pathological aggregation, neuropathological findings reveal that this balance is not maintained equally across disease contexts, as evidenced by the absence of TDP-43 inclusions in certain forms of ALS. Specifically, Tan et al. found TDP-43 inclusions in the brains and spinal cords of sporadic ALS (sALS) and non-mutant superoxide dismutase 1 (SOD1) familial ALS (fALS), but not in two mutant SOD1 cases [39]. Subsequent clinicopathological studies have shown that ALS resulting from FUS mutations likewise exhibits FUS-positive, TDP-43-negative inclusions [40,41]. Their findings were corroborated by Mackenzie et al., who found TDP-43 inclusions in sALS and non-SOD1 fALS neurons, but not in SOD1 fALS tissue [36,42]. A question was then put forward as to what was making TDP-43 inclusions common in sALS and not as easily observable in fALS tissues [36]. This prompted another group to explore if TDP-43, on its own, was prone to aggregation or sequestered by other aggregated components, thus simply making it a “marker of disease” [43].

In their study, Johnson et al. demonstrated that recombinant TDP-43, without interacting cofactors, rapidly formed aggregates after a lag phase of approximately 5–10 min, as indicated by increased solution turbidity and a corresponding increase in pellet size following centrifugation [43]. Identical conditions were applied to control proteins (e.g., BSA, soybean trypsin inhibitor, creatine kinase, and GFP), but no aggregation was observed, indicating that TDP-43 is inherently prone to aggregation [43]. Using a yeast mutant model, the same group showed that reported ALS mutations (e.g., Q331K, M337V, Q343R) accelerated aggregation in vitro and increased the formation of aggregates in vivo.

We acknowledge that many of the studies discussed in this review rely on cellular, *C. elegans*, mouse, and zebrafish models. Interspecies differences in TDP-43 sequence, splicing targets, lifespan, and proteostatic capacity are likely to shift the threshold at which a second hit occurs, but do not fundamentally change the underlying cascade logic. Moreover, human neurons, particularly long-lived projection neurons, exhibit distinct vulnerabilities that could further accentuate specific stages of the cascade, such as clearance failure.

In summary, these findings highlight that TDP-43 is inherently prone to aggregation, with its sequence and domains already predisposing it to self-assembly. Consequently, even in its native state, the protein exhibits oligomerization hotspots that could ultimately manifest as pathological inclusions. This underscores why the protein is heavily dependent on stringent and continuous quality control. Two distinct degradatory and clearance pathways help clear aberrant, misfolded proteins and the aggregates they form. However, several factors can disrupt their principal components, which will be discussed in the succeeding sections.

## 3. TDP-43 Evasion of Cellular Clearance Systems

### 3.1. Dual-Pathway Control: The Proteasome and Autophagy Governing TDP-43

The degradation of aberrant TDP-43 depends on two central quality control systems: (1) the Ubiquitin-Proteasome System (UPS), which degrades soluble, ubiquitinated monomers, and (2) the Autophagy-Lysosome Pathway (ALP), which sequesters larger aggregates in double-membrane vesicles for lysosomal fusion and protease-mediated degradation [44]. For larger, aggregated proteins, the ALP takes over, assisted by adaptor proteins such as sequestosome 1 (p62) and NBR1, which bind to polyubiquitin chains and simultaneously interact with LC3 [44]. Polyubiquitin tags are not limited to UPS, but are also recognized by the ALP via p62 and NBR1 [45,46,47].

While ubiquitin tagging can guide proteins toward either proteasomal or autophagic degradation, the exact functional partitioning between the two systems remained unclear for TDP-43. Scotter et al. (2014) explored this gap, ultimately demonstrating that the UPS processes soluble TDP-43, whereas the ALP handles aggregates to facilitate their clearance [48]. More importantly, they found that cellular macroaggregates of TDP-43 are reversible when both systems are functional, and further postulated that a “second hit” in either of these two systems drives the pathological cascade of TDP-43 aggregation in ALS and FTD [48]. In their study, stable cell lines were generated in which expression of different HA-tagged TDP-43 variants could be induced with doxycycline. The expressed constructs were: (1) wild-type (HA-TDP-43-WT), (2) ΔNLS-truncated mutant (HA-TDP-43-ΔNLS), and (3) C-terminal fragment (aa 181–414) (HA-TDP-43-CTF). Both the localization and expression of the constructs were then observed following treatment with autophagy inhibitors (e.g., 3-methyladenine (3MA), bafilomycin (Baf)) or UPS inhibitors (e.g., MG132 and epomoxin) [48].

The results revealed a distinct separation of TDP-43 processing between the two degradation systems. Autophagy was shown to be the leading player in clearing TDP-43 CTFs, whereas the UPS primarily targeted soluble WT/ΔNLS TDP-43 [48]. Inhibiting UPS resulted in the pronounced accumulation and aggregation of soluble TDP-43 species. The researchers used a doxycycline (DOX)-inducible system in SH-SY5Y and HEK293 cells to express HA-tagged TDP-43 [48]. Applying MG-132, a reversible UPS inhibitor, resulted in the formation of large, cytoplasmic, and ubiquitin-positive aggregates [48]. This was followed by a 48 h washout period to remove the MG-132, and end-point imaging revealed that the cells were devoid of TDP-43 and p62 aggregates, even when TDP-43 expression continued via DOX induction [48]. The cells were then immediately transferred to fresh medium containing 3MA (which blocks the initial stages of autophagy) and bafilomycin (Baf; which prevents the fusion of autophagosomes with lysosomes) to block autophagy upon restoration of UPS function [48]. The authors then observed that macroaggregates still disassembled into smaller, discrete particles (microaggregates), demonstrating that the initial fragmentation of larger aggregates is an autophagy-independent step. Nevertheless, in the presence of autophagy inhibitors, these microaggregates persisted in the cells. Conversely, the activation of autophagy with trehalose or lithium chloride (LiCl) enhanced CTF degradation in separate experiments, validating its principal role in clearing TDP-43 aggregates [48]. When these findings are viewed holistically, they build the following picture: (1) degradation of soluble TDP-43 species is mainly via the UPS, (2) once protein oligomerization is initiated, these species will have become too large to enter the proteasome pore, requiring the intervention of another pathway (i.e., autophagy) [48]. Notably, the study demonstrates that only when both systems are functional can the cells completely reverse TDP-43 macroaggregate formation, further substantiating the cooperative interplay between the UPS and autophagy pathways. An important limitation worth noting here is the biological cost of clearance, where 69.3% of aggregate-laden cells died during the 15 h clearance period [48]. Reversibility was only observed in the minority of cells that managed to survive the initial aggregate burden.

These findings are consistent with previously published work, which shows that different degradation pathways process distinct TDP-43 species. Specifically, Wang et al. (2010) showed that the truncated form of TDP-43 (TDP-25) was more reliant on the autophagy pathway [49]. Furthermore, Urushitani et al. (2010) revealed the synergistic effect of the proteasome and autophagosome in clearing polyubiquitinated TDP-43, corroborating the dual-pathway requirement [50]. Additionally, proteasome inhibition has been shown to drive the cytoplasmic accumulation and aggregation of neuronal TDP-43, supporting the central role of the UPS in degrading soluble TDP-43 species [51]. Conversely, the role of autophagy in clearing truncated or aggregation-prone species was demonstrated through the rapamycin-mediated activation of the pathway, resulting in the rescue of mislocalized TDP-43 and reduction in CTF accumulation in the cytosol [52]. The tight, cooperative interplay between the UPS and ALP systems is critical for TDP-43 proteostasis, providing the mechanistic rationale for how a ‘second hit’ to either system triggers the pathological cascade observed in ALS and FTLD.

### 3.2. CTD Mutations Increase Structural Stability and Resistance

The domains of TDP-43 are indeed critical in influencing its folding behavior, particularly the highly disordered CTD. Other domains, such as the NTD, were demonstrated to have the capability of counteracting this intrinsic disorder through specific structural arrangements that provide spatial separation of the CTD, thereby attenuating uncontrolled folding and aggregation. However, certain conditions, such as the presence of mutations, can enhance the stability of its folding, potentially shifting the balance toward more aggregation-prone conformations.

Particularly, mutations (G298S, Q331K, M337V) in the CTD introduce structural modifications in its primary sequence, which potentially lead to impairment in lysosomal or proteasomal degradation, as well as favoring irreversible aggregation over normal LLPS, thereby decreasing TDP-43’s susceptibility to degradation [53,54,55,56,57]. An early study by Ling et al. (2010) [58] explored the effect of these CTD mutations on the half-life of TDP-43. Here, tetracycline-inducible wild-type and mutant genes were expressed using a cytomegalovirus (CMV) promoter in isogenic cell lines. By randomly cycling the cells through brief incubation with [^35^S]methionine/cysteine, newly synthesized proteins were radiolabeled and subsequently monitored over time to measure their half-lives [58]. The results showed that mutant proteins were degraded two to four times more slowly than wt-TDP-43 [58]. Moreover, immunoprecipitation results indicated stronger interaction between endogenous wt-FUS/TLS and TDP-43 mutants, suggesting that mutation-induced CTD stabilization not only affects folding but also enhances cross-interaction with FUS/TLS [58].

The structural disruptions resulting from these mutations (e.g., A321G, A321V, Q331K, M337V) were analyzed in detail using NMR spectroscopy, simulation, and microscopy, revealing a CTD subregion that transiently folds into a helix, thereby mediating TDP-43 phase separation [54]. The mutations disrupt this phase separation by inhibiting the interaction and destabilizing the α-helical structure. Notably, A321G and Q331K eliminated the phase separation of the CTD, while M337V decreased solution turbidity by approximately 50% [54]. Removing the region containing the α-helical structure and the mutations (Δ321–343), and the helix-disrupting mutation (A326P) resulted in the loss of phase separation, suggesting the participation of this helical region is essential in LLPS [54]. At low concentrations, the truncated CTD as well as the mutant CTD remained soluble but easily formed insoluble aggregates when their concentrations were increased, demonstrating that this helical region is necessary for stabilizing the liquid-like state of TDP-43 [54]. Zeng et al. (2024) similarly showed the disruptive effect of M337V in the same region with their own simulations, corroborating the above results [56]. Taken together, these findings suggest that residues 321–343 form a critical region that promotes and maintains the reversibility of TDP-43 LLPS through its intermolecular helix-helix interactions with the same region of other TDP-43 molecules. However, mutations within this segment disrupt these interactions, destabilizing LLPS and thereby shifting the equilibrium toward irreversible aggregation.

### 3.3. Mutations Can Also Increase Vulnerability to Enzymatic Cleavage

So far, we have established that mutations in the CTD are a disruptive force that favors the formation of irreversible aggregates. However, some mutations paradoxically render TDP-43 more vulnerable to protease-mediated cleavage. Investigations into the role of calpains in promoting the cleavage of mutant TDP-43 proteins were prompted by the hypothesis that the downregulation of adenosine deaminase acting on RNA 2 (ADAR2) enzymes enhances the generation of TDP-43 pathology [30]. ADAR2 enzymes convert the adenosine found in the Q/R site of GluA2 (the protein subunit of α-amino-3-hydroxy-5-methylisoxazole-4-propionic acid or AMPA receptors) pre-mRNA to inosine, facilitating the impermeability of AMPA receptors to calcium ions (Ca^2+^) [59,60]. However, the motor neurons of patients with sporadic ALS have reduced levels of ADAR2, resulting in leaky AMPA receptors due to the decreased processing of GluA2 pre-mRNA [61,62]. The reduced ADAR2 leads to increased uptake of Ca^2+^, which can be neurotoxic. The increased Ca^2+^ uptake likewise prompted Yamashita et al. to investigate the proportional effect of Ca^2+^-dependent proteases on TDP-43 pathology, and they found an increased generation of C-terminal fragments (CTFs), with A315T and M337V TDP-43 mutants being cleaved faster than wild-type TDP-43 (Figure 1) [30]. The group further demonstrated that knocking out AR2 in mice led to TDP-43 mislocalization and cleavage, resulting from calpain activation triggered by Ca^2+^ AMPA receptors. In contrast, AR2res (AR2-restored) mice, which expressed Ca^2+^-impermeable AMPA receptors even in the absence of AR2, showed no TDP-43 pathology or calpain activation, confirming that Ca^2+^ influx is the trigger of TDP-43 pathology [30].

A separate study by Chiang et al. (2016) demonstrated that the D169G mutation exhibits higher thermal stability compared to wild-type TDP-43 and is cleaved by caspase-3 more efficiently, resulting in the production of more CTFs (TDP-35) in neuroblastoma cells [31]. In vitro, caspase-3 cleaves the D169G mutant approximately two-fold more efficiently than wt-TDP-43 (40.3 ± 3.8% vs. 20 ± 3.4% cleaved N RRM12 after 2 h) [31]. The D169G mutation is located in the RRM1 domain and induces a local conformational change in a α-turn, resulting in increased hydrophobic interactions within the RRM1 core (Figure 1) [31]. The resulting more rigid conformation possibly exposes more caspase-3 cleavage sites to the solvent, thereby enhancing cleavage efficiency. Based on these findings, the effects of TDP-43 cleavage appear paradoxical at first glance; cleavage is generally expected to be a biologically favorable process that facilitates the clearance of TDP-43 deposits from cells. However, this is not always the case; the cleavage site and the properties of the resulting fragments are critically important, as specific cleavage fragments can, in fact, exacerbate aggregation. Specifically, the generation of CTFs has been shown to promote aggregation due to their increased tendency to self-associate and nucleate TDP-43 oligomers [63,64,65,66,67,68]. In this case, cleavage is not inherently beneficial; however, the type and properties of the resulting fragments dictate the likelihood of their clearance or persistence within the cytoplasm.

### 3.4. Proteasomal Evasion: Failure to Recognize and Process Soluble Species

As established in the section above, the UPS is the primary pathway for processing soluble TDP-43 monomers. The UPS and the autophagy-lysosome pathways have distinct roles, and the latter cannot compensate for the failure of the former to process TDP-43 soluble species. This functional specialization means that the persistence of pathological TDP-43 in the cytoplasm of affected neurons is a critical indication that the UPS is somehow overwhelmed or circumvented in disease states. Proteasomal evasion, therefore, is not just a consequence of aggregate size exceeding the 20S core aperture but involves specific molecular factors that impede efficient substrate recognition and digestion. The failure of the UPS is linked primarily to two factors that direct the toxic shift towards TDP-43 oligomerization and aggregation: (1) intrinsic substrate characteristics that disrupt ubiquitination, and (2) extrinsic factors that compromise proteasome function.

#### 3.4.1. Intrinsic Substrate Factors That Influence UPS Effectivity

The contribution of each lysine residue to the ubiquitination of TDP-43 was explored by Hans et al. (2018) [69] using single and multiple-site lysine mutants. Their study revealed that the four lysine residues in the CTF, spanning residues 193–414, do not contribute to ubiquitination; however, all four lysines needed to be removed for complete suppression of ubiquitination [69]. Additionally, the substitution of Lys-408 enhanced the pathological phosphorylation of Ser-409/410, a marker commonly observed in TDP-43 pathology [21,69,70,71]. However, these effects were not observed when extended to full-length TDP-43. Mutagenesis of Lys-84 and Lys-95 in the NLS reinforced that these residues had a more significant role in ubiquitination compared to any of the CTD lysines [69]. Overall, these findings suggest that TDP-43 harbors multiple, functionally redundant ubiquitination sites distributed across its domains. Thus, inefficient TDP-43 ubiquitination likely stems not from a lack of modification sites but from other intrinsic properties of the protein.

The disordered nature of the CTD promotes aggregation and phase separation, potentially masking ubiquitination sites and interfering with UPS interaction [10,72]. Specifically, the misfolding of the protein and the formation of aggregates make ubiquitination sites less accessible to E3 ligases and other components of the UPS, thereby decreasing the efficiency of recognizing the tagged proteins and their subsequent degradation. Moreover, the accumulated CTFs in the cytoplasm have been shown to interact with proteasome assembly proteins, thereby disrupting the formation of the proteasome complex and resulting in inefficient degradation [73]. TDP-43 aggregates were also demonstrated to sequester ubiquitin and UPS components into inclusions, depleting the pool of free ubiquitin needed for tagging aberrant proteins [74]. Thus, the impairment of UPS activity creates a vicious cycle, accelerating TDP-43 aggregation and leading to cellular dysfunction and neurotoxicity. While the CTD has been extensively documented for its role in aggregation and contribution to UPS evasion, NTD conformation was also suggested to influence TDP-43 aggregation dynamics, albeit through more subtle and less frequent mechanisms.

Early NMR and CS-Rosetta modeling by Qin et al. (2014) suggested that the NTD of TDP-43 can adopt a ubiquitin-like fold in slow equilibrium with a highly disordered state, and that binding to single-stranded DNA (ssDNA) favors the shift toward the folded conformation, leading to the proposal that nucleic-acid binding stabilizes the NTD, protecting against aggregation [75]. Subsequent high-resolution NMR analysis by Mompeán et al. (2016), which incorporated far more extensive NOE and dynamics data, showed that the isolated NTD (residues 1–77) forms a stably folded domain whose topology more closely represents the axin-1 Dix domain, with no unfolded population under a broad range of conditions [76]. Here, the disordered 78–102 segment, rather than the folded NTD core, was enough for binding DNA oligonucleotides, leading the authors to argue that nucleic acid binding is unlikely needed to stabilize the NTD fold per se and that the physiological relevance of folding NTD dynamics remains unclear [76].

#### 3.4.2. Extrinsic Factors That Modulate UPS Activity

Complementing these intrinsic properties are extrinsic factors, which can modulate or intensify the disruption of UPS activity. The 26S proteasome consists of a 20S core particle, a hollow complex composed of four heteroheptameric rings with an α_7_-α_7_-α_7_-α_7_ orientation [77,78]. A small gate or flap (the N-termini of α-subunits) folds over the 13Å aperture of the core subunit, preventing unregulated access to catalytic sites within [79]. The opening of the gate is controlled by the binding of the HbYX motif from the 19S ATPases to intersubunit pockets on the α-ring, creating a conformational change that permits substrate entry [80]. However, pathologic proteins were shown to bind to these intersubunit pockets, rather than engaging the active site to open the core subunit (as in HbYX), which further stabilizes the closed conformation and impairs gate activation altogether.

This principle of inhibition by extrinsic oligomers has been demonstrated for Aα, α-synuclein, and mutant huntingtin, which share a common structural epitope that interacts with the intersubunit pockets of the 20S core particle to inhibit gate opening allosterically [81]. Notably, spherical TDP-43 oligomers have been detected in the hippocampal and frontal cortical tissue of FTD-TDP patients using oligomeric TDP-43-specific antibodies that bind to a different epitope [82]. However, a study by Riemenschneider et al. expressed GFP-TDP-25 (residues 220–414) in primary neurons, showing (via cryo-electron tomography and subtomogram) an eight-fold increase of 26S proteasomes inside TDP-25 inclusions compared to the surrounding cytoplasm. They further demonstrated that these sequestered proteasomes exhibited substrate-processing conformations of the 19S regulatory particle, and ground-state particles were nearly absent, strongly indicating that these proteasomes were stalled mid-cycle due to the interaction [83]. Additionally, an extra density in the 3D average that was not accounted for by known proteasome subunits was observed and regarded as a substrate or adaptor protein bound to the 19S regulatory subunit [83]. Thus, these findings suggest that the CTF of TDP-43 is more likely to interact with the 19S regulatory subunit of the proteasome rather than the 20S core subunit. Other studies pointed to TDP-43 interacting with proteasome assembly factors PSMG2 and PSD13, reducing the pool of fully assembled proteasomes [73]. Nevertheless, by analogy to A11-positive Aα, α-synuclein, and huntingtin oligomers, it is possible that certain TDP-43 oligomeric strains could engage the 20S core and also disturb gate dynamics, but this remains to be tested with purified proteasomes [81].

Taken together, these findings underscore the central role of a common structural epitope found in protein oligomers that arise from common neurodegenerative diseases in inhibiting proteasomal function. This suggests that the specific oligomer type is secondary to the structural conformation causing the interference; thus, targeting this unique shape may restore proteasomal activity. Equally important is considering the significance of the potential impact that the comorbid expression of these oligomers will have on the failed clearance of TDP-43 aggregates, as observed in some pathologies [84].

Additionally, other sources of stress can further burden the UPS, particularly the proteasome. Endoplasmic reticulum (ER) stress, for instance, has been shown to compromise proteasomal degradation, as treatment with various ER stressors delayed the turnover of ER-localized reporters and led to the subtle but consistent accumulation of multiple nuclear/cytosolic ones [85]. A similar accumulation was observed in transgenic mice expressing UPS reporter substrates following induction of ER stress. Furthermore, ER-stressed cells failed to clear UBB^+1^, an aberrant ubiquitin variant associated with conformational diseases, resulting in impaired UPS activity [85].

Oxidative stress represents another primary consideration that undermines UPS efficiency. Some studies have shown that mild oxidative stress can transiently enhance UPS activity, whereas chronic or severe oxidative stress can progressively compromise both ubiquitin-conjugating enzymes and the proteasome [86,87]. Key residues in the proteasomal core and its regulatory subunits can undergo oxidative structural changes, rendering the complex functionally impaired [88,89]. Oxidation of critical active sites can likewise reduce proteolytic efficiency [90,91]. In addition, cysteine residues in E1, E2, and E3 enzymes, which are responsible for ubiquitin conjugation, are susceptible to oxidation, potentially compromising their activity [86,87]. Nevertheless, in some cases, protein degradation can still proceed independently of ubiquitination [86]. Ultimately, the excessive accumulation of aberrant proteins can obstruct the proteasomal gate, creating a vicious cycle in which progressive impairment of degradation pathways further enhances proteotoxic stress [89,91].

Alongside ER and oxidative stress, other chronic cellular insults such as hypoxia, hyperglycemia associated with diabetes, and persistent neuroinflammation were also reported to contribute to this systemic burden on the UPS [92,93,94,95,96]. Ultimately, the interplay of physical occlusion, resulting from chemical modifications, and chronic cellular stress compromises the biological efficiency of the UPS, allowing soluble TDP-43 to persist and rapidly transition into aggregation-prone species, which subsequently burden the autophagy-lysosomal pathway.

### 3.5. Autophagic Evasion

**Figure 2:**
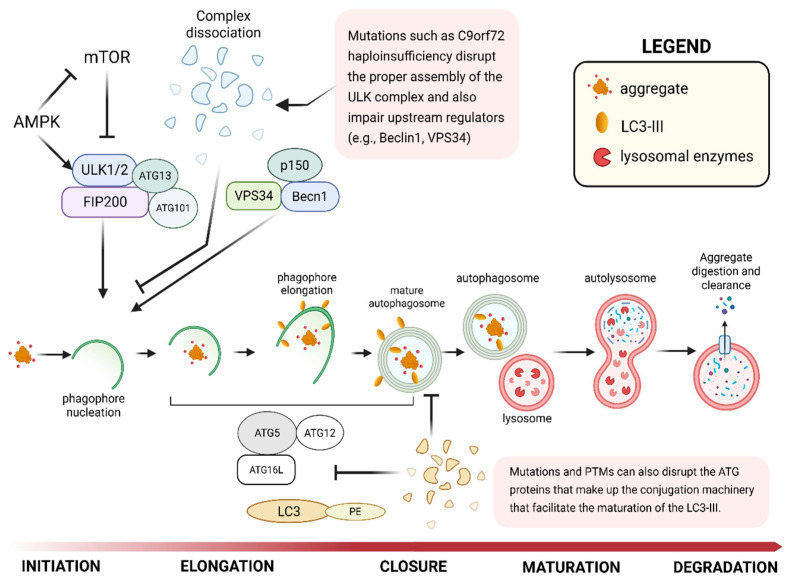
Schematic of the autophagy–lysosome pathway (ALP) in TDP-43 clearance, highlighting key initiation, cargo recognition, and lysosomal degradation steps. Abbreviations: UPS, ubiquitin–proteasome system; ALP, autophagy–lysosome pathway; ULK1, UNC-51–like kinase 1; ATG, autophagy-related protein; LC3, microtubule-associated protein 1 light chain 3; p150, regulatory scaffold subunit (VPS15/PIK3R4) of the class III PI3K complex.

Building on what was introduced earlier, protein processing by the UPS can occasionally be inaccurate and exacerbated by the stressors mentioned above. Hence, misfolded TDP-43 oligomers that slip past the UPS could coalesce into larger aggregates that the ALP processes. Autophagy is the cell’s main bulk degradation pathway, which sequesters large, insoluble aggregates, damaged organelles, and aberrant proteins into specialized vesicles called autophagosomes, which then fuse with lysosomes, organelles that contain hydrolytic enzymes for degradation (Figure 2) [97,98,99]. Given its high processing capacity, the ALP should, in theory, be capable of processing and clearing the protein aggregates that escape the UPS. However, the unopposed formation of TDP-43 aggregates is associated with the dysfunction of the ALP at several critical stages, resulting from a failed recognition, impaired influx, and compromised downstream clearance, which will be discussed next.

#### 3.5.1. Impaired Autophagy Initiation and Maturation

Due to the wider variety, larger size, and greater stability of its protein substrates, the ALP requires several more groups of receptors, chaperones, and enzymes to facilitate effective degradation and clearance. The pathway begins with the formation of the initiation (ULK1/ATG1) complex, which is composed of ULK1, ATG13, ATG101, and FIP200, and induces autophagosome formation (Figure 2) [100,101]. Now, C9orf72 (chromosome 9 ORF 72) is a well-documented protein with mutations associated with ALS and FTD [102], and one study has implicated it in Ras-related protein Rab 1a (Rab1a)-dependent trafficking of ULK1 to sites that initiate autophagy [103]. Here, the reduction in C9orf72 expression in HeLa or HEK293 cells via RNA interference directed against different regions of C9orf72, as well as in rat primary cortical neurons using miRNA, resulted in an attenuated increase in autophagosomes in siRNA-treated cells compared to the untreated cells following Torin1 treatment (an autophagy initiator) [103]. Further, the absence of changes in FIP200 puncta from basal levels in C9orf72 knockdown cells and neurons following Torin1 treatment demonstrated the critical role of C9orf72 in the translocation of the ULK1 protein complex to the phagophore [103]. These results suggest that C9orf72 plays a crucial role in regulating autophagy initiation by binding to Rab1a and directing the trafficking of ULK1 during the process. Therefore, failed TDP-43 clearance in ALS and FTD may be associated with the inability to initiate autophagosome formation resulting from C9orf72 haploinsufficiency.

Following the assembly of the initiation (ULK1) complex is the formation of the phosphoinositide 3-kinase (PI3K) complex made up of VPS34, Beclin1, ATG13, and p150/VPS15. The PI3K complex is required to synthesize phosphatidyl inositol 3-phosphate (PI3P), and recruit other effector proteins (e.g., WIPI2, DFCP1) to facilitate membrane expansion and maturation into an autophagosome (Figure 2) [104,105]. This growth is sustained by the Atg12-Atg5/Atg16 complex and the LC3 lipidation system [106]. The impairment of VPS34 was shown to disrupt autophagosome formation and endosomal maturation, which are critical pathways for TDP-43 clearance [107,108]. The loss of VPS34 or Beclin1 (as demonstrated in knockdown experiments) also destabilized other components of the complex, including ATG14, creating a feedback loop for dysfunction [108]. These findings likewise demonstrate that defective TDP-43 clearance may be associated with destabilization of the PI3K complex, resulting in failed autophagosome maturation.

#### 3.5.2. Failed Cargo Recognition

Other components, such as autophagy receptors (e.g., p62/SQSTM1, NBR1, and OPTN), which recognize and bind various protein cargoes to direct them towards the autophagosome, could also be disrupted due to structural mutations (Figure 2). Specifically, mutations linked to ALS/FTD in SQSTM1 interfere with the protein’s biological function by reducing its phosphorylation by ULK1 and TBK1, inhibiting its binding to ubiquitin, and effectively impeding the clearance of TDP-43 aggregates [109]. The accumulation of p62 is also considered a hallmark of disrupted autophagy in TDP-43 proteinopathies. It is correlated with the loss of TDP-43 function, suggesting that TDP-43 plays a regulatory role in the ALP through a feedback loop [110,111]. Optineurin (OPTN) is another selective autophagy receptor involved in the clearance of ubiquitinated proteins and damaged mitochondria [112,113,114]. OPTN mutations, such as the ALS-associated E478G variant, prevent the degradation of TDP-43 by disrupting autophagosome formation [115]. OPTN interacts with the kinase TBK1, and inhibition of this interaction adversely affects ALP downstream processes [112,113,116].

#### 3.5.3. Compromised Downstream Clearance

Another essential component of the ALP is LC3-II (lipidated LC3), a microtubule-associated protein involved in sealing the membrane of the mature autophagosome (Figure 2). This protein is also recognized as an indicator of increased autophagosome formation or interrupted autophagosome-lysosome fusion. Separate studies revealed the colocalization of TDP-43 puncta with LC3-positive autophagic compartments, as well as the LC3-II-positive material and autophagic markers associated with extracellular TDP-43 resulting from interrupted lysosomal-autophagic fusion [117,118]. This coaccumulation of LC3-II with TDP-43 aggregates is a clear indication that failed autophagic processes contribute significantly to TDP-43 deposition within cells.

Finally, essential components that facilitate the fusion of the mature autophagosome with the lysosome comprise the SNARE (Soluble NSF Attachment protein REceptor) complex. These proteins complete the fusion of the autophagic and lysosomal membranes, and are made up of STX17 on the autophagosomes, and SNAP29 (cytosolic Qbc-SNARE) and VAMP8 on lysosomes [119]. Despite the limited studies associating TDP-43 with SNARE protein aberration, the disruption of fusion observed in TDP-43 proteinopathies strongly suggests a connection [120]. STX17 recruits SNAP29, which then results in the binding of VAMP8 to create the fusion-capable SNARE complex [119]. When any of the critical components are disrupted, the fusion of the autophagosome-lysosome complex fails and is reflected by the accumulation of unfused autophagosomes [119,121].

Collectively, these findings firmly establish that a robust and cooperative interplay between the UPS and ALP is crucial for maintaining TDP-43 proteostasis. Given the intrinsic propensity of TDP-43 to misfold and form oligomers, along with the myriad of extrinsic factors that could interfere with the UPS and the inherent complexity and number of regulatory steps of the ALP (Table 1), this dual requirement forms a mechanistic basis to illustrate that just a few destabilizing “second hits” to either system is enough to reduce the clearance of TDP-43 aggregates effectively.

## 4. Dynamics and Systemic Failure Further the Persistence of Pathologic TDP-43

While intrinsic resistance and mechanistic properties facilitate the evasion of aberrant TDP-43 from protein clearance pathways within the nucleus, these are insufficient to account for the relentless progression of aggregation characteristic of neurodegenerative diseases. The transition from transient evasion to a stable, self-propagating mechanism is a result of a combination of several factors, including a shift in the cellular environment. These intrinsic modifications (e.g., mutations) promote aggregation kinetics and, most importantly, systemic failure of the cell’s quality control infrastructure. This section examines downstream mechanisms following successful evasion, where the addition of PTMs promotes nucleation, and the formed aggregate overwhelms both systems, particularly the ALP, driving the process into a self-sustaining toxic cycle.

As misfolded TDP-43 accumulates outside the nucleus, a significant shift in its cellular distribution occurs. The aggregation dynamics involved in this cytoplasmic relocation further favor its sequestration into irreversible aggregates; the factors that drive this process forward will be discussed next.

### 4.1. Shifting the Environment: Nuclear Depletion and Aggregation Kinetics

TDP-43 normally resides and performs its regulatory functions within the nucleus. Still, a small fraction of its concentration is regularly shuttled to the cytoplasm to perform functions in RNA metabolism and regulation of translation [11]. In the nucleus, the RRM domains of TDP-43 bind with high affinity, stabilizing the protein and maintaining it in a soluble state [37,122,123]. One study demonstrated that the removal of the RRM domains using site-directed mutagenesis significantly reduced TDP-43 levels within the nucleoplasm, underscoring the valuable role of RRM domains in TDP-43 nuclear localization [123]. This cooperative binding produces a higher-order assembly on mRNA, facilitating the reduction in intermolecular interactions between the aggregate-prone NTD and CTD, which could result in aggregation [122].

However, once TDP-43 exits the nucleus, multiple factors further reduce its RNA-binding capacity. PTMs, such as the acetylation of lysines (e.g., K136 and K145), disrupt RNA binding and enhance phase separation through the CTD, leading to the formation of insoluble aggregates [10,124]. Acetylation mimicking mutants (K145Q) exhibited insoluble, hyperphosphorylated, and ubiquitinated aggregates [21]. Another mutation (K181E) found in the RRM-linking region also reduced RNA-binding affinity and enhanced aggregate formation [125]. These findings confirm that the solubility and conformational integrity of TDP-43 are significantly dependent on sustained RNA engagement.

Once RNA engagement is weakened, the resulting consequence significantly contributes to the increased formation of TDP-43 aggregates [72,126]. Additional studies have described the formation of anisotropic intranuclear liquid droplets exhibiting HSP70 chaperones, which translates to a state of pathological assembly [127]. Furthermore, RNA-binding-deficient TDP-43 was shown to undergo demixing in stress granules (SGs), where the TDP-43-rich phase has significantly reduced RNA levels compared to the G3BP1-rich phase [128]. G3BP1 (Ras GTPase-activating protein-binding protein 1) functions as a scaffolding protein that initiates stress granule assembly. Together with other RBPs and RNAs, it contributes to the formation of a dynamic, liquid-like phase that constitutes the structural matrix of normal SGs. TDP-43 is also included in this phase, but prolonged oxidative stress and the buildup of misfolded TDP-43 result in its “demixing” from the G3BP1 matrix, creating its own subphase within the granule [128]. Thus, the observed loss of RNA in the TDP-43 phase corroborates that maintaining TDP-43 solubility highly depends on its RNA-binding capability.

For additional context and to avoid confusion with regular liquid-liquid phase separation, intra-condensate demixing is an additional phase separation event within an existing biomolecular condensate, in which a previously uniform droplet spontaneously segregates into compositionally distinct subphases, such as a TDP-43-enriched core within a stress granule [128]. This additional step is thermodynamically favorable because it allows for maximizing the newly acquired interactions of the TDP-43 molecules while minimizing unfavorable contacts with other stress-granule components, lowering the overall free energy of the condensate [128]. This also increases the local abundance of reactive oligomeric species that can react with functional proteins and contribute to cellular toxicity. More details on the toxic species of TDP-43 are discussed in the following section.

Additionally, Yan et al. (2025) [128] concluded that although the formation of SGs increased TDP-43 concentration above a critical threshold, this alone was insufficient for aggregation. Conversely, when prolonged oxidative stress resulting in cysteine oxidation and structural destabilization within the RRM domain (and increased hydrophobic interactions in the CTD) was the only variable treated, but TDP-43 levels failed to reach the critical threshold in SGs, no aggregation was observed [128]. Therefore, both variables (the crucial concentration of TDP-43 in SGs and prolonged oxidation) are essential for the phase separation of TDP-43 to occur within the granules and form irreversible aggregates with pathological hallmarks.

Mechanistically, RNA binding increases the stability of the RRM domains and inhibits the conformational plasticity of the protein. Molecular dynamics simulations demonstrated that the absence of RNA promotes the tandem RRMs to exhibit enhanced conformational plasticity, particularly RRM1, which displays an increased likelihood of adopting partially unfolded conformations [129]. The interaction with RNA introduces constraints to TDP-43, increasing intradomain stability and inhibiting the structural transitions that lead to uncontrolled aggregation [129].

Moreover, single-molecule tracking revealed a significant reduction in TDP-43 mobility with increasing stress duration, where the effective diffusion coefficients dropped from a baseline of 3 μm^2^/s to 0.75 μm^2^/s after two hours of arsenite-induced stress [130]. This was observed for both free cytoplasmic TDP-43 and TDP-43 sequestered into SGs, suggesting that oligomeric hotspots, which could potentially lead to irreversible aggregates, may result from cytoplasmic TDP-43, not just those already sequestered into SGs.

Finally, the cytoplasm fundamentally lacks the quality control mechanisms that maintain TDP-43 homeostasis and solubility in the nucleus, fostering an environment that predisposes TDP-43 to misfolding and aggregation. Nuclear TDP-43 is delicately retained by forming large RNA-mediated macromolecular complexes that block its cytoplasmic diffusion in a size-dependent manner [37]. These nuclear compartments are also stabilized by RNA binding, oligomerization, and phase separation, where TDP-43 is organized into compartments that maintain its solubility and functionality [131].

Studies have also demonstrated that passive diffusion is the primary force driving TDP-43 cytoplasmic egress, where the protein continuously leaks into the cytoplasm but is actively shuttled back to the nucleus through nucleoporins and import factors [5]. Investigation of the interactome of TDP-43 insoluble aggregates revealed their enrichment in the components of the nuclear pore complex and nucleocytoplasmic machinery, indicating the direct sequestration of these essential karyopherins, leading to the impairment of the nuclear pore complex [38,132]. The result is a feed-forward mechanism in which the increased levels of aberrant cytoplasmic TDP-43 progressively sequester more components, disrupting nuclear import and leading to further accumulation of cytoplasmic TDP-43.

Another study revealed that the monomerization of TDP-43 is key in driving the nuclear export of the protein. Monomeric TDP-43 was demonstrated to preferentially interact with nuclear RNA export factor 1 (Nxf1), compared to its dimeric form [133]. Additionally, the monomerization was shown to be triggered by cellular stresses or dysfunctions (i.e., spliceosomal impairment), compared to physiological conditions, where TDP-43 dimerizes or forms higher-order oligomers to facilitate its nuclear retention and solubility [133].

Taken together, reduced affinity to RNA due to structural mutations or PTMs, reaching critical concentration thresholds within SGs (demixing), prolonged oxidative stress, the absence of quality control mechanisms in the cytoplasm, and the resulting vicious cycle induced by the sequestration of critical importins by the increased egress of aberrant TDP-43 are all significant drivers that produce a self-reinforcing cascade that drive irreversible aggregation.

### 4.2. Toxic Species That Drive TDP-43 Pathology

The propensity of TDP-43 to naturally form aggregates has been described, with its early phase characterized as an oligomeric species that is stable, spherical, and amyloid-like, featuring exposed hydrophobic surfaces [43,82]. These oligomers were reported to have reduced nucleic-acid binding and direct neurotoxicity in neuronal models [82]. Regions in TDP-43 that facilitate its self-aggregation were identified, and candidate peptides targeting these regions were designed to reduce TDP-43 aggregation. Despite identifying two constructs that successfully reduced aggregation in a dose-dependent manner, the reduction in aggregation did not prevent cell death [134]. However, it must be noted that the authors considered only observable aggregates; thus, it is possible that cell death could mainly stem from the toxicity of soluble oligomers, which have much more exposed hydrophobic regions that can interact with functional cellular proteins relative to stable aggregates [135]. Moreover, liquid-like RNP granules in axons become more viscous and less dynamic with ALS-linked mutations, disrupting granule transport and inducing the toxic gain-of-function effect [136]. Finally, cytoplasmic liquid droplets that formed independently of classical SGs were reported to gel up or solidify under stress, which is enough to disrupt nuclear import and deplete nuclear TDP-43, resulting in cell death [38,130]. Given these findings, primary toxic TDP-43 species appear to arise from oligomeric and gel-like assemblies rather than end-stage fibrils, thereby combining direct proteotoxicity with the disruption of RNA metabolism and nucleocytoplasmic transport.

CTFs are also species that play a significant role in TDP-43 toxicity, as they have been characterized as able to seed full-length TDP-43. On their own, they are capable of inducing cell death through a toxic gain-of-function without sequestering their full-length counterpart [65], as well as disrupting proper proteasomal assembly [84]. Together, these findings support a conceptual framework in which soluble oligomers, viscous RNP granules, and CTFs act as the principal toxic species. At the same time, large aggregates may be later consequences that correlate with, but do not strictly determine, neuronal death. Simply put, pathogenicity may be best explained as a combination of misfolded assembly-mediated gain of function and depletion of functional TDP-43 within the nucleus.

### 4.3. The Aggregation Cascade: Acceleration and Propagation Dynamics

Now that we have established the key molecular players involved in the mislocalization of TDP-43, we will discuss more factors that further accelerate the formation of TDP-43 aggregates once the initial stages have been fulfilled. These factors are critical to understand, as they substantially increase the rate of TDP-43 entanglement. Several steps within this process may also serve as therapeutic targets to slow or interrupt the pathological cascade.

#### 4.3.1. PTMs as Structural Destabilizers and Pathological Markers

The key mechanism driving the acceleration of aggregation is the reduction in the energy threshold required for the formation of oligomeric hotspots, as well as the stabilization of the insoluble core of the fibril. One of the mechanisms exploited by the addition of PTMs involves reducing the RNA-binding affinity within the RRM domains.

Acetylation is one PTM recognized for reducing the binding affinity of the RRMs, as discussed in the previous sections. The loss of the interacting RNA further exposes the CTD, significantly lowering the nucleation barrier or phase separation. The acetylation of crucial lysine residues (e.g., K145, K192, and K136) was demonstrated to enhance the tendency for TDP-43 to form solid-like or oligomeric assemblies [16]. This phase separation was described as anisomes, which are nuclear membraneless organelles that act as seeds for aggregation, and are pathologically observed in ALS/FTD tissue [16]. Physiologically, the nucleus fosters an environment that maintains the solubility and homeostasis of TDP-43. Still, the introduction of structural mutations and charge-neutralizing PTMs reduces RNA affinity of nuclear TDP-43, leading to the creation of these membraneless compartments.

Oxidative stress is another mechanism that can structurally modify cysteine residues in the RRMs (e.g., C173, C175, C198, and C244), inducing local unfolding or domain swapping, ultimately resulting in rapid and stable α-sheet-rich oligomers through the formation of intermolecular disulfide bridges [137]. Another PTM is citrullination, which functions similarly by neutralizing the positive charge on arginines, weakening RNA affinity, and favoring the shift to phase separation. These modifications were suggested to drive TDP-43 past the solubility threshold, increasing the rate of oligomer growth and aggregation [138].

As discussed previously, ubiquitination is a critical PTM that tags misfolded TDP-43 for degradation via the ALP. However, due to the impaired cascade reminiscent of ALS and FTD pathologies, ubiquitinated TDP-43 substantially accumulates in the cytoplasm, with the ubiquitin tags acting as markers of failed autophagy [13]. While phosphorylation is consistently observed in ALS/FTD tissues [139,140,141,142], its precise role remains debated, with some evidence suggesting it may initially function as a protective clearance signal before contributing to aggregate insolubility [9,111,140].

#### 4.3.2. Lowering the Nucleation Barrier: Accumulation of Misfolded Monomers Eventually Leads to Uncontrolled Aggregation and Systemic Collapse

The net balance between TDP-43 aggregate formation and clearance modulates whether its deposition or removal from the cell is favored. For instance, as discussed earlier, the half-life of the soluble phase of TDP-43 significantly determines its clearance rate, owing to the increased structural stability of the misfolded form. Wild-type TDP-43 has been shown to have a half-life ranging from 4 to 12 h in immortalized cells; however, it has also been reported to last as long as 18 h in primary neurons [143]. Depending on various experimental conditions, the reported values can range from 4 to 34 h [48,58,144,145]. This variability is influenced by a combination of physiological and biological factors, as well as the experimental methods employed. First is subcellular localization, where TDP-43 localized within the nucleus is highly stable due to a less proteolytically active and more chemically homogenous environment [146]. In contrast, cytoplasmic proteins are exposed to more proteases and lysosomes, which constantly sample and remove damaged or misfolded proteins that have accumulated. Second is the type of cell, which showed varying protein stability in different models. Primary human fibroblasts reportedly have half-lives of 4 h, while differentiated Neuro2a cells have a half-life of 12.6 h, and HEK293 and SH-SY5Y cells have half-lives ranging from 30 to 33 h [48,58,145]. This variability results from differences in proteasome and lysosome-autophagy capacities in cells, which directly affect TDP-43 turnover [58,147]. Moreover, endogenous proteins are reportedly often found to have shorter half-lives (e.g., 4 h) compared to studies using inducible expression or transgenes in established cell lines, which typically range between 12 and 34 h [48,58,144], suggesting that primary cells may have more efficient degradation dynamics than immortalized lab cultures [58]. Finally, the type of tag used for visualizing and purifying the protein may impact its stability [48,58]. While some argue that the use of large tags does not affect normal function, others have observed that EGFP-tagged fragments can exhibit different steady-state levels and functional effects compared to smaller epitope tags [48,58]. However, in the presence of mutations, the half-lives of monomeric TDP-43 generally increase due to the conferred resistance to temperature-induced unfolding, aggregation, and degradation, resulting in elevated steady-state TDP-43 concentrations within the cell [148]. PTMs, such as citrullination, acetylation, and oxidation, also contribute to pathogenesis by destabilizing the interaction of TDP-43 monomers with RNA, promoting the misfolded state, and rendering the protein more resistant to proteolytic processing by the UPS [16,137,138].

This elevation of steady-state concentrations is critical because it leads to the initial accumulation of misfolded aberrant monomers that escape UPS processing, subsequently serving as potential substrates for the next onslaught. While the rate-limiting step in TDP-43 pathogenesis is the elevated nucleation barrier that monomers must overcome to form stable and insoluble fibrils, this persistent monomer accumulation increases the likelihood of intermolecular interactions, leading to the formation of small soluble oligomers [148,149]. The resulting oligomers act as potent seeds that facilitate surmounting the thermodynamic barrier, leading to the recruitment and conversion of the remaining soluble monomers into insoluble aggregates at a significantly accelerated rate [26,150,151,152]. This abrupt shift from spontaneous nucleation (lag phase) to seeded growth (exponential phase) corresponds to the kinetic inflection point that leads to uncontrollable self-propagation. Finally, aggregation kinetics can also be promoted extrinsically through cross-seeding. Oligomers of co-pathologies, such as α-synuclein, can further reduce the lag time by acting as heterologous molecular scaffolds to support the growing TDP-43 oligomers [82,153].

**Figure 3:**
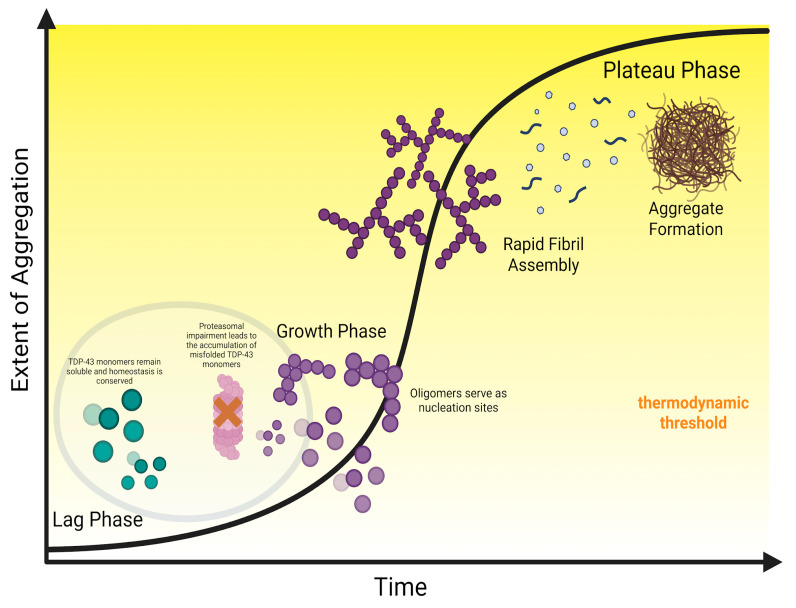
The Kinetic Cascade of TDP-43.

Taken together, the complex interplay between these variables indicates a debilitating kinetic cascade, summarized visually in Figure 3. Pathological mutations or PTMs (e.g., citrullination, oxidation, acetylation) weaken RNA binding, resulting in a pool of proteolysis-resistant monomers that the UPS could not process. The accumulated monomers eventually bypass the nucleation barrier, further facilitated by the formation of catalytic sites, such as stress granules, that foster LLPS irreversibility. The resulting oligomers (including C-terminal fragments and co-pathologies) function as seeds that lower the thermodynamic barrier, driving the pathology from gradual accumulation to exponential fibril growth. Following the full maturation of the aggregates, the rate at which the fibrils form overwhelms the sequestration capacity of the ALP, leading to a feed-forward loop where the impairment of nuclear import mechanisms further saturates dysfunctional TDP-43 in the cytoplasm, triggering the onset of a pathological state.

Each kinetic stage of the cascade can be characterized by specific biomarker levels of the proteins produced by the species. For instance, depleted ubiquitin levels and substantial differences in unassembled proteasomal core subunits could indicate failed assembly of proteasomal complexes or functional impairment. Early-stage TDP-43 oligomer levels could also be assessed using conformation-specific antibodies [82]. In contrast, p62, LC3, and other lysosomal markers could be considered, as well as imaging, to evaluate the assembly of the ALP and autophagic flux in later stages of the cascade. Characterization of each stage with unique biomarker profiles could enable more accurate targeting by interventions and a better understanding of underlying processes.

### 4.4. Buffered Nucleus, Toxic Cytoplasm: Resolving the TDP-43 Aggregation Paradox

The apparent paradox, where TDP-43 aggregates are predominantly cytosolic despite their steady-state nuclear concentration being much higher, can be clarified by considering the different buffering capacities of these compartments. In the nucleus, high-affinity RNA binding and the formation of large ribonucleoprotein assemblies, nuclear bodies, and anisotropic intranuclear droplets (anisomes) maintain TDP-43 in dynamic, liquid-like states, and keep its concentration of free, aggregate-competent monomers low [38,127]. By contrast, once monomerization and RNA disengagement enable TDP-43 entry into the cytoplasm, the protein reaches an environment with weaker RNA buffering, fewer dedicated chaperone elements, and more chronic stressors, driving phase separation in stress granules and demixed cytoplasmic droplets that could rapidly solidify into irreversible aggregates [154].

In this view, intranuclear anisomes represent a high-density but still regulated ‘buffer’ for RNA-free TDP-43, whereas the cytoplasm functions like a sink where the quality-control capacity is lower, nucleocytoplasmic transport is progressively impaired, and aggregates become trapped as hyperphosphorylated, ubiquitinated inclusions [38].

## 5. Conclusions

The pathogenicity of TDP-43 originates not only from its intrinsic tendency to aggregate but is compounded by external factors that allow it to evade the cell’s quality control mechanisms and progress towards systemic collapse. The clearance of TDP-43 aggregates requires the participation of various chaperones, ligases, and enzymes to retain TDP-43 homeostasis as it is continuously shuttled between the nucleus and the cytoplasm. However, these can be disturbed by mutations, structural modifications by PTMs, and chronic cellular stress, potentially initiating the pathologic cascade.

Crucially, distinct TDP-43 species are processed by separate pathways: the UPS handles misfolded soluble monomers, and the ALP sequesters and clears the larger insoluble aggregates. This functional partitioning underscores that these two systems are not fully redundant. Consequently, the ALP cannot effectively compensate for UPS failure and vice versa. Synergistic function is required to effectively clear the proteins, meaning a ‘second hit’ compromising either system is sufficient to trigger the pathological cascade.

Motor neurons, with their long axons, high metabolic demands, and heavy cargo trafficking, require a high proteostatic load to meet these biological requirements [155]. This increased load reflects the continuous need to maintain protein quality control in large, metabolically active neurons, which show high autophagic and proteasomal flux even under baseline conditions [155]. At the same time, neurons possess limited regenerative capacity and rely heavily on the fidelity of RNA-splicing networks to maintain cellular homeostasis [156]. Factors such as mutations, chronic stress, and aging can easily trigger minor perturbations that can propagate magnified effects [156]. The mentioned factors, along with the unique biological demands of neurons, effectively lower the threshold at which the cascade becomes pathologic, hence their disproportionate vulnerability compared to other cells [155].

Disruptions to the UPS originate from two convergent pathways: the intrinsic aggregation propensity of monomeric TDP-43 and external insults, particularly chronic oxidative stress, hypoxia, and neuroinflammation. These insults can compromise critical UPS components, such as ubiquitin-conjugating enzymes and proteasomal core subunits, preventing their assembly into functional degradation complexes. This vulnerability demonstrates that TDP-43 is not the sole player in initiating the pathological process. Nevertheless, a pool of misfolded monomers arises, priming the cellular environment for the next stage: the growth of oligomers, marked by the phase separation of TDP-43 in stress granules. These oligomeric assemblies act as seeds, enabling TDP-43 to circumvent the energy barrier for uncontrolled self-assembly. This relentless feed-forward cycle marks the exponential phase of the cascade, which eventually overwhelms the autophagy-lysosome pathway with the rapid growth of fibrils and aggregates. Additionally, although the toxic role of TDP-43 oligomeric species is well-documented, the inhibitory effect of these species on the proteasome remains unclear, presenting an interesting avenue for future research.

Due to the complexity and multiplicity of the pathways involved, the roles of some processes in the aggravation or suppression of TDP-43 clearance remain undefined. Future studies should validate the kinetic contribution of co-pathologies, such as amyloid beta, a-synuclein, and huntingtin, as well as the role of CTFs in sequestering other essential but understudied components of the UPS or ALP. A growing number of studies are also focusing on the role of protein oligomers, which are considered more toxic than fibrils, and how the pathological cascade can be quelled more effectively if targeted in its earlier stages. Finally, a consensus is yet to be reached on whether the accumulation of TDP-43 aggregates is the primary toxic driver or if the loss of protein function is the principal culprit, rendering aggregates a physical, inert manifestation of the dysfunction.

Regardless of the specific toxic species, these findings strongly suggest that TDP-43 pathogenesis is a collapse in kinetic regulation. Future interventions must extend beyond simple clearance strategies and explore options for breaking the feed-forward loops that drive the transition from regulatable monomers to self-propagating oligomers. Our synthesis reiterates that simply upregulating bulk clearance will unlikely yield improvements in TDP-43 cellular homeostasis. Instead, future interventions need to be aligned with kinetic inflection points in TDP-43 misfolding, phase separation, and clearance failure. In this framework, TDP-43-targeting antisense nucleotides, molecular scaffolds that bring aberrant TDP-43 and E3 ligase closer together for proteasomal processing, and modulators of chaperone or LLPS behavior are being developed to target monomers and early-stage oligomers in the cascade [58,157,158,159]. On the other hand, UPS and ALP enhancers, which act as ligands to activate autophagosome or lysosome biogenesis, are strategies that target later stages where aggregates have accumulated [160]. In place of an exhaustive catalog of candidate therapies, our goal here is to clarify how these diverse strategies can be interpreted within this kinetic landscape, complementing more detailed therapeutic reviews.

## Figures and Tables

**Table 1:** Intrinsic and extrinsic factors that promote TDP-43 aggregation and evasion of cellular clearance.

Category	Factor/Type	Representative Examples or Mechanisms
Intrinsic	Disordered CTD and aggregation cores	Glycine-rich CTD, residues 279–360/282–360 forming amyloid cores, and steric-zipper/LARKS motifs that accelerate assembly [28,29].
	Pathogenic CTD mutations	G298S, Q331K, M337V, and related variants that slow degradation, destabilize LLPS, and enhance irreversible aggregation and CTF accumulation [54,56,58].
	Aberrant PTMs	Cysteine oxidation, pathological phosphorylation, and cleavage (e.g., TDP-43/TDP-35) destabilize RNA binding and generate aggregation-prone fragments [31,35,50].
	Oligomerization hotspots	NTD/RRM interfaces that form oligomers; structural changes that shift from protective oligomerization to pathogenic seeding states [33,34,35,43].
Extrinsic	UPS impairment	Proteasome inhibition, sequestration of proteasome subunits and ubiquitin, and disrupted assembly (e.g., via TDP-43 CTF–PSMG2/PSMD13 interactions) [69,83].
	ALP dysfunction	Defective ULK1–ATG initiation, impaired cargo recognition by p62/NBR1, and lysosomal compromise that limit aggregate clearance [35,50].
	Cellular stressors	Chronic ER and oxidative stress, hypoxia, hyperglycemia, and neuroinflammation burden UPS/ALP capacity and promote proteotoxic cycles [94,95,96,97,98].
	Co-pathologies and oligomer crosstalk	Coexisting Aβ, α-synuclein, or huntingtin oligomers that compete for proteostasis resources and may further inhibit proteasomal function [82].

## Data Availability

No new data were created or analyzed in this study. Data sharing is not applicable to this article.

## Abbreviations

AD	Alzheimer’s disease
ADAR2	Adenosine deaminase acting on RNA 2
ALP	Autophagy–lysosome pathway
ALS	Amyotrophic lateral sclerosis
AMPA	α-Amino-3-hydroxy-5-methyl-4-isoxazolepropionic acid (glutamate receptor)
ASO	Antisense oligonucleotide
Baf	Bafilomycin A1
BSA	Bovine serum albumin
C9orf72	Chromosome 9 open reading frame 72
Ca²⁺	Calcium ion
CMV	Cytomegalovirus
CTD	C-terminal domain
CTF	C-terminal fragment (of TDP-43)
DNA	Deoxyribonucleic acid
ER	Endoplasmic reticulum
fALS	Familial ALS
FTD	Frontotemporal dementia
FTLD	Frontotemporal lobar degeneration
FTLD-TDP	FTLD with TDP-43 pathology
FTLD-tau	FTLD with tau pathology
FUS	Fused in sarcoma (RNA-binding protein)
GFP	Green fluorescent protein
GluA2	Glutamate receptor 2 (AMPA receptor subunit)
GRR	Glycine-rich region
HbYX	Conserved hydrophobic–tyrosine–any amino acid C-terminal motif of certain ATPases
hnRNP	Heterogeneous nuclear ribonucleoprotein
LARKS	Low-complexity aromatic-rich kinked segments
LATE	Limbic-predominant age-related TDP-43 encephalopathy
LCD	Low-complexity domain
LC3	Microtubule-associated protein 1 light chain 3
LiCl	Lithium chloride
LLPS	Liquid–liquid phase separation
mRNA	Messenger RNA
NES	Nuclear export signal
NLS	Nuclear localization signal
NTD	N-terminal domain
p62	Sequestosome 1 (SQSTM1)
PTM	Post-translational modification
RRM	RNA recognition motif
RNA	Ribonucleic acid
sALS	Sporadic ALS
SG	Stress granule
SOD1	Superoxide dismutase 1
SSDNA	Single-stranded DNA
TARDBP	Gene encoding TDP-43
TDP-43	Transactive response DNA-binding protein of 43 kDa
TDP-25/TDP-35	Truncated TDP-43 fragments of approximately 25/35 kDa
UBB+1	Frameshifted ubiquitin B variant
UPS	Ubiquitin–proteasome system

## References

[1] Song J. (2024). Molecular Mechanisms of Phase Separation and Amyloidosis of ALS/FTD-Linked FUS and TDP-43. Aging Dis..

[2] Tollervey J.R., Curk T., Rogelj B., Briese M., Cereda M., Kayikci M., König J., Hortobágyi T., Nishimura A.L., Zupunski V. (2011). Characterizing the RNA Targets and Position-Dependent Splicing Regulation by TDP-43. Nat. Neurosci..

[3] Bedja-Iacona L., Richard E., Marouillat S., Brulard C., Alouane T., Beltran S., Andres C.R., Blasco H., Corcia P., Veyrat-Durebex C. (2024). Post-Translational Variants of Major Proteins in Amyotrophic Lateral Sclerosis Provide New Insights into the Pathophysiology of the Disease. Int. J. Mol. Sci..

[4] Versluys L., Ervilha Pereira P., Schuermans N., De Paepe B., De Bleecker J.L., Bogaert E., Dermaut B. (2022). Expanding the TDP-43 Proteinopathy Pathway from Neurons to Muscle: Physiological and Pathophysiological Functions. Front. Neurosci..

[5] Pinarbasi E.S., Cağatay T., Fung H.Y.J., Li Y.C., Chook Y.M., Thomas P.J. (2018). Active Nuclear Import and Passive Nuclear Export Are the Primary Determinants of TDP-43 Localization. Sci. Rep..

[6] Doll S.G., Meshkin H., Bryer A.J., Li F., Ko Y.-H., Lokareddy R.K., Gillilan R.E., Gupta K., Perilla J.R., Cingolani G. (2022). Recognition of the TDP-43 Nuclear Localization Signal by Importin α1/β. Cell Rep..

[7] Ederle H., Funk C., Abou-Ajram C., Hutten S., Funk E.B.E., Kehlenbach R.H., Bailer S.M., Dormann D. (2018). Nuclear Egress of TDP-43 and FUS Occurs Independently of Exportin-1/CRM1. Sci. Rep..

[8] Ayala Y.M., De Conti L., Avendaño-Vázquez S.E., Dhir A., Romano M., D’Ambrogio A., Tollervey J., Ule J., Baralle M., Buratti E. (2011). TDP-43 Regulates Its mRNA Levels through a Negative Feedback Loop. EMBO J..

[9] Gruijs da Silva L.A., Simonetti F., Hutten S., Riemenschneider H., Sternburg E.L., Pietrek L.M., Gebel J., Dötsch V., Edbauer D., Hummer G. (2022). Disease-linked TDP-43 hyperphosphorylation suppresses TDP-43 condensation and aggregation. EMBO J..

[10] Garcia Morato J., Hans F., von Zweydorf F., Feederle R., Elsässer S.J., Skodras A.A., Gloeckner C.J., Buratti E., Neumann M., Kahle P.J. (2022). Sirtuin-1 Sensitive Lysine-136 Acetylation Drives Phase Separation and Pathological Aggregation of TDP-43. Nat. Commun..

[11] Birsa N., Bentham M.P., Fratta P. (2020). Cytoplasmic Functions of TDP-43 and FUS and Their Role in ALS. Semin. Cell Dev. Biol..

[12] Neumann M., Sampathu D.M., Kwong L.K., Truax A.C., Micsenyi M.C., Chou T.T., Bruce J., Schuck T., Grossman M., Clark C.M. (2006). Ubiquitinated TDP-43 in Frontotemporal Lobar Degeneration and Amyotrophic Lateral Sclerosis. Science.

[13] Prasad A., Bharathi V., Sivalingam V., Girdhar A., Patel B.K. (2019). Molecular Mechanisms of TDP-43 Misfolding and Pathology in Amyotrophic Lateral Sclerosis. Front. Mol. Neurosci..

[14] Nelson P.T., Dickson D.W., Trojanowski J.Q., Jack C.R., Boyle P.A., Arfanakis K., Rademakers R., Alafuzoff I., Attems J., Brayne C. (2019). Limbic-Predominant Age-Related TDP-43 Encephalopathy (LATE): Consensus Working Group Report. Brain.

[15] Budini M., Romano V., Quadri Z., Buratti E., Baralle F.E. (2015). TDP-43 Loss of Cellular Function through Aggregation Requires Additional Structural Determinants beyond Its C-Terminal Q/N Prion-like Domain. Hum. Mol. Genet..

[16] Keating S.S., Bademosi A.T., San Gil R., Walker A.K. (2023). Aggregation-Prone TDP-43 Sequesters and Drives Pathological Transitions of Free Nuclear TDP-43. Cell. Mol. Life Sci..

[17] Suk T.R., Part C.E., Zhang J.L., Nguyen T.T., Heer M.M., Caballero-Gómez A., Grybas V.S., McKeever P.M., Nguyen B., Ali T. (2025). A Stress-Dependent TDP-43 SUMOylation Program Preserves Neuronal Function. Mol. Neurodegener..

[18] Verde E.M., Antoniani F., Mediani L., Secco V., Crotti S., Ferrara M.C., Vinet J., Sergeeva A., Yan X., Hoege C. (2025). SUMO2/3 Conjugation of TDP-43 Protects against Aggregation. Sci. Adv..

[19] Lee S., Kwon Y., Kim S., Jo M., Jeon Y.-M., Cheon M., Lee S., Kim S.R., Kim K., Kim H.-J. (2020). The Role of HDAC6 in TDP-43-Induced Neurotoxicity and UPS Impairment. Front. Cell Dev. Biol..

[20] Gimenez J., Spalloni A., Cappelli S., Ciaiola F., Orlando V., Buratti E., Longone P. (2023). TDP-43 Epigenetic Facets and Their Neurodegenerative Implications. Int. J. Mol. Sci..

[21] Cohen T.J., Hwang A.W., Restrepo C.R., Yuan C.-X., Trojanowski J.Q., Lee V.M.Y. (2015). An Acetylation Switch Controls TDP-43 Function and Aggregation Propensity. Nat. Commun..

[22] Kumar S.T., Nazarov S., Porta S., Maharjan N., Cendrowska U., Kabani M., Finamore F., Xu Y., Lee V.M.-Y., Lashuel H.A. (2023). Seeding the Aggregation of TDP-43 Requires Post-Fibrillization Proteolytic Cleavage. Nat. Neurosci..

[23] Franzmann T.M., Alberti S. (2019). Prion-like Low-Complexity Sequences: Key Regulators of Protein Solubility and Phase Behavior. J. Biol. Chem..

[24] Staderini T., Bigi A., Mongiello D., Cecchi C., Chiti F. (2022). Biophysical Characterization of Full-Length TAR DNA-Binding Protein (TDP-43) Phase Separation. Protein Sci..

[25] Verde E.M., Secco V., Ghezzi A., Mandrioli J., Carra S. (2025). Molecular Mechanisms of Protein Aggregation in ALS-FTD: Focus on TDP-43 and Cellular Protective Responses. Cells.

[26] Babinchak W.M., Haider R., Dumm B.K., Sarkar P., Surewicz K., Choi J.-K., Surewicz W.K. (2019). The Role of Liquid-Liquid Phase Separation in Aggregation of the TDP-43 Low-Complexity Domain. J. Biol. Chem..

[27] Guenther E.L., Cao Q., Trinh H., Lu J., Sawaya M.R., Cascio D., Boyer D.R., Rodriguez J.A., Hughes M.P., Eisenberg D.S. (2018). Atomic Structures of TDP-43 LCD Segments and Insights into Reversible or Pathogenic Aggregation. Nat. Struct. Mol. Biol..

[28] Kumar V., Wahiduzzaman, Prakash A., Tomar A.K., Srivastava A., Kundu B., Lynn A.M., Imtaiyaz Hassan M. (2019). Exploring the Aggregation-Prone Regions from Structural Domains of Human TDP-43. Biochim. Biophys. Acta Proteins Proteom..

[29] Arseni D., Chen R., Murzin A.G., Peak-Chew S.Y., Garringer H.J., Newell K.L., Kametani F., Robinson A.C., Vidal R., Ghetti B. (2023). TDP-43 Forms Amyloid Filaments with a Distinct Fold in Type A FTLD-TDP. Nature.

[30] Yamashita T., Hideyama T., Hachiga K., Teramoto S., Takano J., Iwata N., Saido T.C., Kwak S. (2012). A Role for Calpain-Dependent Cleavage of TDP-43 in Amyotrophic Lateral Sclerosis Pathology. Nat. Commun..

[31] Chiang C.-H., Grauffel C., Wu L.-S., Kuo P.-H., Doudeva L.G., Lim C., Shen C.-K.J., Yuan H.S. (2016). Structural Analysis of Disease-Related TDP-43 D169G Mutation: Linking Enhanced Stability and Caspase Cleavage Efficiency to Protein Accumulation. Sci. Rep..

[32] Cohen T.J., Hwang A.W., Unger T., Trojanowski J.Q., Lee V.M.Y. (2012). Redox signalling directly regulates TDP-43 via cysteine oxidation and disulphide cross-linking: Oxidative stress regulates TDP-43. EMBO J..

[33] Afroz T., Hock E.-M., Ernst P., Foglieni C., Jambeau M., Gilhespy L.A.B., Laferriere F., Maniecka Z., Plückthun A., Mittl P. (2017). Functional and Dynamic Polymerization of the ALS-Linked Protein TDP-43 Antagonizes Its Pathologic Aggregation. Nat. Commun..

[34] Jiang L.-L., Xue W., Hong J.-Y., Zhang J.-T., Li M.-J., Yu S.-N., He J.-H., Hu H.-Y. (2017). The N-Terminal Dimerization Is Required for TDP-43 Splicing Activity. Sci. Rep..

[35] Wang A., Conicella A.E., Schmidt H.B., Martin E.W., Rhoads S.N., Reeb A.N., Nourse A., Ramirez Montero D., Ryan V.H., Rohatgi R. (2018). A Single N-Terminal Phosphomimic Disrupts TDP-43 Polymerization, Phase Separation, and RNA Splicing. EMBO J..

[36] Rothstein J.D. (2007). TDP-43 in Amyotrophic Lateral Sclerosis: Pathophysiology or Patho-Babel?. Ann. Neurol..

[37] Dos Passos P.M., Hemamali E.H., Mamede L.D., Hayes L.R., Ayala Y.M. (2024). RNA-Mediated Ribonucleoprotein Assembly Controls TDP-43 Nuclear Retention. PLoS Biol..

[38] Gasset-Rosa F., Lu S., Yu H., Chen C., Melamed Z., Guo L., Shorter J., Da Cruz S., Cleveland D.W. (2019). Cytoplasmic TDP-43 DE-Mixing Independent of Stress Granules Drives Inhibition of Nuclear Import, Loss of Nuclear TDP-43, and Cell Death. Neuron.

[39] Tan C.-F., Eguchi H., Tagawa A., Onodera O., Iwasaki T., Tsujino A., Nishizawa M., Kakita A., Takahashi H. (2007). TDP-43 Immunoreactivity in Neuronal Inclusions in Familial Amyotrophic Lateral Sclerosis with or without SOD1 Gene Mutation. Acta Neuropathol..

[40] King A., Troakes C., Smith B., Nolan M., Curran O., Vance C., Shaw C.E., Al-Sarraj S. (2015). ALS-FUS Pathology Revisited: Singleton FUS Mutations and an Unusual Case with Both a FUS and TARDBP Mutation. Acta Neuropathol. Commun..

[41] Blair I.P., Williams K.L., Warraich S.T., Durnall J.C., Thoeng A.D., Manavis J., Blumbergs P.C., Vucic S., Kiernan M.C., Nicholson G.A. (2010). FUS Mutations in Amyotrophic Lateral Sclerosis: Clinical, Pathological, Neurophysiological and Genetic Analysis. J. Neurol. Neurosurg. Psychiatry.

[42] Mackenzie I.R.A., Bigio E.H., Ince P.G., Geser F., Neumann M., Cairns N.J., Kwong L.K., Forman M.S., Ravits J., Stewart H. (2007). Pathological TDP-43 Distinguishes Sporadic Amyotrophic Lateral Sclerosis from Amyotrophic Lateral Sclerosis with SOD1 Mutations. Ann. Neurol..

[43] Johnson B.S., Snead D., Lee J.J., McCaffery J.M., Shorter J., Gitler A.D. (2009). TDP-43 Is Intrinsically Aggregation-Prone, and Amyotrophic Lateral Sclerosis-Linked Mutations Accelerate Aggregation and Increase Toxicity. J. Biol. Chem..

[44] Kubota H. (2009). Quality Control against Misfolded Proteins in the Cytosol: A Network for Cell Survival. J. Biochem..

[45] Pankiv S., Clausen T.H., Lamark T., Brech A., Bruun J.-A., Outzen H., Øvervatn A., Bjørkøy G., Johansen T. (2007). P62/SQSTM1 Binds Directly to Atg8/LC3 to Facilitate Degradation of Ubiquitinated Protein Aggregates by Autophagy. J. Biol. Chem..

[46] Kirkin V., Lamark T., Sou Y.-S., Bjørkøy G., Nunn J.L., Bruun J.-A., Shvets E., McEwan D.G., Clausen T.H., Wild P. (2009). A Role for NBR1 in Autophagosomal Degradation of Ubiquitinated Substrates. Mol. Cell.

[47] Johansen T., Lamark T. (2011). Selective Autophagy Mediated by Autophagic Adapter Proteins. Autophagy.

[48] Scotter E.L., Vance C., Nishimura A.L., Lee Y.-B., Chen H.-J., Urwin H., Sardone V., Mitchell J.C., Rogelj B., Rubinsztein D.C. (2014). Differential Roles of the Ubiquitin Proteasome System and Autophagy in the Clearance of Soluble and Aggregated TDP-43 Species. J. Cell Sci..

[49] Wang X., Fan H., Ying Z., Li B., Wang H., Wang G. (2010). Degradation of TDP-43 and Its Pathogenic Form by Autophagy and the Ubiquitin-Proteasome System. Neurosci. Lett..

[50] Urushitani M., Sato T., Bamba H., Hisa Y., Tooyama I. (2010). Synergistic Effect between Proteasome and Autophagosome in the Clearance of Polyubiquitinated TDP-43. J. Neurosci. Res..

[51] van Eersel J., Ke Y.D., Gladbach A., Bi M., Götz J., Kril J.J., Ittner L.M. (2011). Cytoplasmic Accumulation and Aggregation of TDP-43 upon Proteasome Inhibition in Cultured Neurons. PLoS ONE.

[52] Caccamo A., Majumder S., Deng J.J., Bai Y., Thornton F.B., Oddo S. (2009). Rapamycin Rescues TDP-43 Mislocalization and the Associated Low Molecular Mass Neurofilament Instability. J. Biol. Chem..

[53] Sampognaro P.J., Arya S., Knudsen G.M., Gunderson E.L., Sandoval-Perez A., Hodul M., Bowles K., Craik C.S., Jacobson M.P., Kao A.W. (2023). Mutations in α-Synuclein, TDP-43 and Tau Prolong Protein Half-Life through Diminished Degradation by Lysosomal Proteases. Mol. Neurodegener..

[54] Conicella A.E., Zerze G.H., Mittal J., Fawzi N.L. (2016). ALS Mutations Disrupt Phase Separation Mediated by α-Helical Structure in the TDP-43 Low-Complexity C-Terminal Domain. Structure.

[55] Li H.-R., Chen T.-C., Hsiao C.-L., Shi L., Chou C.-Y., Huang J.-R. (2018). The Physical Forces Mediating Self-Association and Phase-Separation in the C-Terminal Domain of TDP-43. Biochim. Biophys. Acta Proteins Proteom..

[56] Zeng J., Tang Y., Dong X., Li F., Wei G. (2024). Influence of ALS-Linked M337V Mutation on the Conformational Ensembles of TDP-43321-340 Peptide Monomer and Dimer. Proteins.

[57] Hallegger M., Chakrabarti A.M., Lee F.C.Y., Lee B.L., Amalietti A.G., Odeh H.M., Copley K.E., Rubien J.D., Portz B., Kuret K. (2021). TDP-43 Condensation Properties Specify Its RNA-Binding and Regulatory Repertoire. Cell.

[58] Ling S.-C., Albuquerque C.P., Han J.S., Lagier-Tourenne C., Tokunaga S., Zhou H., Cleveland D.W. (2010). ALS-Associated Mutations in TDP-43 Increase Its Stability and Promote TDP-43 Complexes with FUS/TLS. Proc. Natl. Acad. Sci. USA.

[59] Kwak S., Kawahara Y. (2005). Deficient RNA Editing of GluR2 and Neuronal Death in Amyotropic Lateral Sclerosis. J. Mol. Med..

[60] Kawahara Y., Ito K., Sun H., Aizawa H., Kanazawa I., Kwak S. (2004). Glutamate Receptors: RNA Editing and Death of Motor Neurons. Nature.

[61] Aizawa H., Sawada J., Hideyama T., Yamashita T., Katayama T., Hasebe N., Kimura T., Yahara O., Kwak S. (2010). TDP-43 Pathology in Sporadic ALS Occurs in Motor Neurons Lacking the RNA Editing Enzyme ADAR2. Acta Neuropathol..

[62] Hideyama T., Yamashita T., Aizawa H., Tsuji S., Kakita A., Takahashi H., Kwak S. (2012). Profound Downregulation of the RNA Editing Enzyme ADAR2 in ALS Spinal Motor Neurons. Neurobiol. Dis..

[63] Nonaka T., Kametani F., Arai T., Akiyama H., Hasegawa M. (2009). Truncation and Pathogenic Mutations Facilitate the Formation of Intracellular Aggregates of TDP-43. Hum. Mol. Genet..

[64] Prasad A., Sivalingam V., Bharathi V., Girdhar A., Patel B.K. (2018). The Amyloidogenicity of a C-Terminal Region of TDP-43 Implicated in Amyotrophic Lateral Sclerosis Can Be Affected by Anions, Acetylation and Homodimerization. Biochimie.

[65] Zhang Y.-J., Xu Y.-F., Cook C., Gendron T.F., Roettges P., Link C.D., Lin W.-L., Tong J., Castanedes-Casey M., Ash P. (2009). Aberrant Cleavage of TDP-43 Enhances Aggregation and Cellular Toxicity. Proc. Natl. Acad. Sci. USA.

[66] Shimonaka S., Nonaka T., Suzuki G., Hisanaga S.-I., Hasegawa M. (2016). Templated Aggregation of TAR DNA-Binding Protein of 43 kDa (TDP-43) by Seeding with TDP-43 Peptide Fibrils. J. Biol. Chem..

[67] Nonaka T., Masuda-Suzukake M., Arai T., Hasegawa Y., Akatsu H., Obi T., Yoshida M., Murayama S., Mann D.M.A., Akiyama H. (2013). Prion-like Properties of Pathological TDP-43 Aggregates from Diseased Brains. Cell Rep..

[68] Saini A., Chauhan V.S. (2011). Delineation of the Core Aggregation Sequences of TDP-43 C-Terminal Fragment. ChemBioChem.

[69] Hans F., Eckert M., von Zweydorf F., Gloeckner C.J., Kahle P.J. (2018). Identification and Characterization of Ubiquitinylation Sites in TAR DNA-Binding Protein of 43 kDa (TDP-43). J. Biol. Chem..

[70] Tomé S.O., Vandenberghe R., Ospitalieri S., Van Schoor E., Tousseyn T., Otto M., von Arnim C.A.F., Thal D.R. (2020). Distinct Molecular Patterns of TDP-43 Pathology in Alzheimer’s Disease: Relationship with Clinical Phenotypes. Acta Neuropathol. Commun..

[71] Wang P., Wander C.M., Yuan C.-X., Bereman M.S., Cohen T.J. (2017). Acetylation-Induced TDP-43 Pathology Is Suppressed by an HSF1-Dependent Chaperone Program. Nat. Commun..

[72] Pérez-Berlanga M., Wiersma V.I., Zbinden A., De Vos L., Wagner U., Foglieni C., Mallona I., Betz K.M., Cléry A., Weber J. (2023). Loss of TDP-43 Oligomerization or RNA Binding Elicits Distinct Aggregation Patterns. EMBO J..

[73] Yin P., Bai D., Zhu L., Deng F., Guo X., Li B., Chen L., Li S., Li X.-J. (2021). Cytoplasmic TDP-43 Impairs the Activity of the Ubiquitin-Proteasome System. Exp. Neurol..

[74] Farrawell N.E., McAlary L., Lum J.S., Chisholm C.G., Warraich S.T., Blair I.P., Vine K.L., Saunders D.N., Yerbury J.J. (2020). Ubiquitin Homeostasis Is Disrupted in TDP-43 and FUS Cell Models of ALS. iScience.

[75] Qin H., Lim L.-Z., Wei Y., Song J. (2014). TDP-43 N Terminus Encodes a Novel Ubiquitin-like Fold and Its Unfolded Form in Equilibrium That Can Be Shifted by Binding to ssDNA. Proc. Natl. Acad. Sci. USA.

[76] Mompeán M., Romano V., Pantoja-Uceda D., Stuani C., Baralle F.E., Buratti E., Laurents D.V. (2016). The TDP-43 N-Terminal Domain Structure at High Resolution. FEBS J..

[77] Voges D., Zwickl P., Baumeister W. (1999). The 26S Proteasome: A Molecular Machine Designed for Controlled Proteolysis. Annu. Rev. Biochem..

[78] Groll M., Ditzel L., Löwe J., Stock D., Bochtler M., Bartunik H.D., Huber R. (1997). Structure of 20S Proteasome from Yeast at 2.4Å Resolution. Nature.

[79] Groll M., Bajorek M., Köhler A., Moroder L., Rubin D.M., Huber R., Glickman M.H., Finley D. (2000). A Gated Channel into the Proteasome Core Particle. Nat. Struct. Biol..

[80] Smith D.M., Chang S.-C., Park S., Finley D., Cheng Y., Goldberg A.L. (2007). Docking of the Proteasomal ATPases’ Carboxyl Termini in the 20S Proteasome’s Alpha Ring Opens the Gate for Substrate Entry. Mol. Cell.

[81] Thibaudeau T.A., Anderson R.T., Smith D.M. (2018). A Common Mechanism of Proteasome Impairment by Neurodegenerative Disease-Associated Oligomers. Nat. Commun..

[82] Fang Y.-S., Tsai K.-J., Chang Y.-J., Kao P., Woods R., Kuo P.-H., Wu C.-C., Liao J.-Y., Chou S.-C., Lin V. (2014). Full-Length TDP-43 Forms Toxic Amyloid Oligomers That Are Present in Frontotemporal Lobar Dementia-TDP Patients. Nat. Commun..

[83] Riemenschneider H., Guo Q., Bader J., Frottin F., Farny D., Kleinberger G., Haass C., Mann M., Hartl F.U., Baumeister W. (2022). Gel-like Inclusions of C-terminal Fragments of TDP-43 Sequester Stalled Proteasomes in Neurons. EMBO Rep..

[84] Jiang L.-L., Zhang X.-L., Hu H.-Y. (2024). Co-Aggregation of TDP-43 with Other Pathogenic Proteins and Their Co-Pathologies in Neurodegenerative Diseases. Int. J. Mol. Sci..

[85] Menéndez-Benito V., Verhoef L.G.G.C., Masucci M.G., Dantuma N.P. (2005). Endoplasmic Reticulum Stress Compromises the Ubiquitin-Proteasome System. Hum. Mol. Genet..

[86] Shringarpure R., Grune T., Mehlhase J., Davies K.J.A. (2003). Ubiquitin Conjugation Is Not Required for the Degradation of Oxidized Proteins by Proteasome. J. Biol. Chem..

[87] Pickering A.M., Davies K.J.A. (2012). Degradation of Damaged Proteins: The Main Function of the 20S Proteasome. Prog. Mol. Biol. Transl. Sci..

[88] Grune T., Merker K., Sandig G., Davies K.J.A. (2003). Selective Degradation of Oxidatively Modified Protein Substrates by the Proteasome. Biochem. Biophys. Res. Commun..

[89] Stadtman E.R. (2006). Protein Oxidation and Aging. Free Radic. Res..

[90] Davies K.J. (2001). Degradation of Oxidized Proteins by the 20S Proteasome. Biochimie.

[91] Grune T., Reinheckel T., Davies K.J. (1997). Degradation of Oxidized Proteins in Mammalian Cells. FASEB J..

[92] Svikle Z., Peterfelde B., Sjakste N., Baumane K., Verkauskiene R., Jeng C.-J., Sokolovska J. (2022). Ubiquitin-Proteasome System in Diabetic Retinopathy. PeerJ.

[93] Shruthi K., Reddy S.S., Reddy G.B. (2017). Ubiquitin-Proteasome System and ER Stress in the Retina of Diabetic Rats. Arch. Biochem. Biophys..

[94] Broca C., Varin E., Armanet M., Tourrel-Cuzin C., Bosco D., Dalle S., Wojtusciszyn A. (2014). Proteasome dysfunction mediates high glucose-induced apoptosis in rodent beta cells and human islets. PLoS ONE.

[95] Goetzke C.C., Ebstein F., Kallinich T. (2021). Role of Proteasomes in Inflammation. J. Clin. Med..

[96] Alfaro E., Díaz-García E., García-Tovar S., Zamarrón E., Mangas A., Galera R., López-Collazo E., García-Rio F., Cubillos-Zapata C. (2022). Upregulated Proteasome Subunits in COVID-19 Patients: A Link with Hypoxemia, Lymphopenia and Inflammation. Biomolecules.

[97] Ravikumar B., Sarkar S., Davies J.E., Futter M., Garcia-Arencibia M., Green-Thompson Z.W., Jimenez-Sanchez M., Korolchuk V.I., Lichtenberg M., Luo S. (2010). Regulation of Mammalian Autophagy in Physiology and Pathophysiology. Physiol. Rev..

[98] Ryter S.W., Cloonan S.M., Choi A.M.K. (2013). Autophagy: A Critical Regulator of Cellular Metabolism and Homeostasis. Mol. Cells.

[99] Nixon R.A., Rubinsztein D.C. (2024). Mechanisms of Autophagy-Lysosome Dysfunction in Neurodegenerative Diseases. Nat. Rev. Mol. Cell Biol..

[100] Noda N.N., Fujioka Y. (2015). Atg1 Family Kinases in Autophagy Initiation. Cell. Mol. Life Sci..

[101] Zachari M., Ganley I.G. (2017). The Mammalian ULK1 Complex and Autophagy Initiation. Essays Biochem..

[102] Gao J., Douglas A.G.L., Chalitsios C.V., Scaber J., Talbot K., Turner M.R., Thompson A.G. (2025). Neurodegenerative Disease in C9orf72 Repeat Expansion Carriers: Population Risk and Effect of UNC13A. Brain.

[103] Webster C.P., Smith E.F., Bauer C.S., Moller A., Hautbergue G.M., Ferraiuolo L., Myszczynska M.A., Higginbottom A., Walsh M.J., Whitworth A.J. (2016). The C9orf72 Protein Interacts with Rab1a and the ULK1 Complex to Regulate Initiation of Autophagy. EMBO J..

[104] Fracchiolla D., Chang C., Hurley J.H., Martens S. (2020). A PI3K-WIPI2 Positive Feedback Loop Allosterically Activates LC3 Lipidation in Autophagy. J. Cell Biol..

[105] Nähse V., Raiborg C., Tan K.W., Mørk S., Torgersen M.L., Wenzel E.M., Nager M., Salo V.T., Johansen T., Ikonen E. (2023). ATPase Activity of DFCP1 Controls Selective Autophagy. Nat. Commun..

[106] Wu Y., Wang H., Xu H. (2025). Autophagy-Lysosome Pathway in Insulin & Glucagon Homeostasis. Front. Endocrinol..

[107] Liu G., Coyne A.N., Pei F., Vaughan S., Chaung M., Zarnescu D.C., Buchan J.R. (2017). Endocytosis Regulates TDP-43 Toxicity and Turnover. Nat. Commun..

[108] Itakura E., Kishi C., Inoue K., Mizushima N. (2008). Beclin 1 Forms Two Distinct Phosphatidylinositol 3-Kinase Complexes with Mammalian Atg14 and UVRAG. Mol. Biol. Cell.

[109] Deng Z., Lim J., Wang Q., Purtell K., Wu S., Palomo G.M., Tan H., Manfredi G., Zhao Y., Peng J. (2020). ALS-FTLD-Linked Mutations of SQSTM1/p62 Disrupt Selective Autophagy and NFE2L2/NRF2 Anti-Oxidative Stress Pathway. Autophagy.

[110] Bose J.K., Huang C.-C., Shen C.-K.J. (2011). Regulation of Autophagy by Neuropathological Protein TDP-43. J. Biol. Chem..

[111] Brady O.A., Meng P., Zheng Y., Mao Y., Hu F. (2011). Regulation of TDP-43 Aggregation by Phosphorylation and p62/SQSTM1: Regulation of TDP-43 Aggregation by Phosphorylation and p62. J. Neurochem..

[112] Qiu Y., Wang J., Li H., Yang B., Wang J., He Q., Weng Q. (2022). Emerging Views of OPTN (optineurin) Function in the Autophagic Process Associated with Disease. Autophagy.

[113] Moore A.S., Holzbaur E.L.F. (2016). Dynamic Recruitment and Activation of ALS-Associated TBK1 with Its Target Optineurin Are Required for Efficient Mitophagy. Proc. Natl. Acad. Sci. USA.

[114] Shen W.-C., Li H.-Y., Chen G.-C., Chern Y., Tu P.-H. (2015). Mutations in the Ubiquitin-Binding Domain of OPTN/optineurin Interfere with Autophagy-Mediated Degradation of Misfolded Proteins by a Dominant-Negative Mechanism. Autophagy.

[115] Maruyama H., Morino H., Ito H., Izumi Y., Kato H., Watanabe Y., Kinoshita Y., Kamada M., Nodera H., Suzuki H. (2010). Mutations of Optineurin in Amyotrophic Lateral Sclerosis. Nature.

[116] Richter B., Sliter D.A., Herhaus L., Stolz A., Wang C., Beli P., Zaffagnini G., Wild P., Martens S., Wagner S.A. (2016). Phosphorylation of OPTN by TBK1 Enhances Its Binding to Ub Chains and Promotes Selective Autophagy of Damaged Mitochondria. Proc. Natl. Acad. Sci. USA.

[117] Erwin A.L., Chang M.L., Fernandez M.G., Attili D., Russ J.E., Sutanto R., Pinarbasi E.S., Bekier M., Brant T.S., Hahn T. (2024). Molecular Visualization of Neuronal TDP43 Pathology in Situ. bioRxiv.

[118] Tanaka Y., Ito S.-I., Honma Y., Hasegawa M., Kametani F., Suzuki G., Kozuma L., Takeya K., Eto M. (2023). Dysregulation of the Progranulin-Driven Autophagy-Lysosomal Pathway Mediates Secretion of the Nuclear Protein TDP-43. J. Biol. Chem..

[119] Hegedűs K., Takáts S., Kovács A.L., Juhász G. (2013). Evolutionarily Conserved Role and Physiological Relevance of a STX17/Syx17 (syntaxin 17)-Containing SNARE Complex in Autophagosome Fusion with Endosomes and Lysosomes. Autophagy.

[120] Xia Q., Wang H., Hao Z., Fu C., Hu Q., Gao F., Ren H., Chen D., Han J., Ying Z. (2016). TDP-43 Loss of Function Increases TFEB Activity and Blocks Autophagosome-Lysosome Fusion. EMBO J..

[121] Dafsari H.S., Schuler J., Schober E., Möller B., Antebi A., Fanto M., Jungbluth H. (2025). The Space-Time Continuum in Neurological Disorders of the Autophagosome-Lysosome Fusion Machinery. Autophagy Rep..

[122] Rengifo-Gonzalez J.C., El Hage K., Clément M.-J., Steiner E., Joshi V., Craveur P., Durand D., Pastré D., Bouhss A. (2021). The Cooperative Binding of TDP-43 to GU-Rich RNA Repeats Antagonizes TDP-43 Aggregation. eLife.

[123] Ayala Y.M., Zago P., D’Ambrogio A., Xu Y.-F., Petrucelli L., Buratti E., Baralle F.E. (2008). Structural Determinants of the Cellular Localization and Shuttling of TDP-43. J. Cell Sci..

[124] Necarsulmer J.C., Simon J.M., Evangelista B.A., Chen Y., Tian X., Nafees S., Marquez A.B., Jiang H., Wang P., Ajit D. (2023). RNA-Binding Deficient TDP-43 Drives Cognitive Decline in a Mouse Model of TDP-43 Proteinopathy. eLife.

[125] Chen H.-J., Topp S.D., Hui H.S., Zacco E., Katarya M., McLoughlin C., King A., Smith B.N., Troakes C., Pastore A. (2019). RRM Adjacent TARDBP Mutations Disrupt RNA Binding and Enhance TDP-43 Proteinopathy. Brain.

[126] Mann J.R., Gleixner A.M., Mauna J.C., Gomes E., DeChellis-Marks M.R., Needham P.G., Copley K.E., Hurtle B., Portz B., Pyles N.J. (2019). RNA Binding Antagonizes Neurotoxic Phase Transitions of TDP-43. Neuron.

[127] Yu H., Lu S., Gasior K., Singh D., Vazquez-Sanchez S., Tapia O., Toprani D., Beccari M.S., Yates J.R., Da Cruz S. (2021). HSP70 Chaperones RNA-Free TDP-43 into Anisotropic Intranuclear Liquid Spherical Shells. Science.

[128] Yan X., Kuster D., Mohanty P., Nijssen J., Pombo-García K., Garcia Morato J., Rizuan A., Franzmann T.M., Sergeeva A., Ly A.M. (2025). Intra-condensate demixing of TDP-43 inside stress granules generates pathological aggregates. Cell.

[129] Ozguney B., Mohanty P., Mittal J. (2024). RNA Binding Tunes the Conformational Plasticity and Intradomain Stability of TDP-43 Tandem RNA Recognition Motifs. Biophys. J..

[130] Streit L., Kuhn T., Vomhof T., Bopp V., Ludolph A.C., Weishaupt J.H., Gebhardt J.C.M., Michaelis J., Danzer K.M. (2022). Stress Induced TDP-43 Mobility Loss Independent of Stress Granules. Nat. Commun..

[131] Scherer N.M., Maurel C., Graus M.S., McAlary L., Richter G., Radford R.A.W., Hogan A., Don E.K., Lee A., Yerbury J. (2024). RNA-Binding Properties Orchestrate TDP-43 Homeostasis through Condensate Formation in Vivo. Nucleic Acids Res..

[132] Chou C.-C., Zhang Y., Umoh M.E., Vaughan S.W., Lorenzini I., Liu F., Sayegh M., Donlin-Asp P.G., Chen Y.H., Duong D.M. (2018). TDP-43 Pathology Disrupts Nuclear Pore Complexes and Nucleocytoplasmic Transport in ALS/FTD. Nat. Neurosci..

[133] Oiwa K., Watanabe S., Onodera K., Iguchi Y., Kinoshita Y., Komine O., Sobue A., Okada Y., Katsuno M., Yamanaka K. (2023). Monomerization of TDP-43 Is a Key Determinant for Inducing TDP-43 Pathology in Amyotrophic Lateral Sclerosis. Sci. Adv..

[134] Liu R., Yang G., Nonaka T., Arai T., Jia W., Cynader M.S. (2013). Reducing TDP-43 Aggregation Does Not Prevent Its Cytotoxicity. Acta Neuropathol. Commun..

[135] Diociaiuti M., Bonanni R., Cariati I., Frank C., D’Arcangelo G. (2021). Amyloid Prefibrillar Oligomers: The Surprising Commonalities in Their Structure and Activity. Int. J. Mol. Sci..

[136] Gopal P.P., Nirschl J.J., Klinman E., Holzbaur E.L.F. (2017). Amyotrophic Lateral Sclerosis-Linked Mutations Increase the Viscosity of Liquid-like TDP-43 RNP Granules in Neurons. Proc. Natl. Acad. Sci. USA.

[137] Rabdano S.O., Izmailov S.A., Luzik D.A., Groves A., Podkorytov I.S., Skrynnikov N.R. (2017). Onset of Disorder and Protein Aggregation due to Oxidation-Induced Intermolecular Disulfide Bonds: Case Study of RRM2 Domain from TDP-43. Sci. Rep..

[138] Saunders C., Rocha-Rangel P., Desai R., Quadri Z., Lui H., Hunt J.B., Liang H., Rogers C., Nash K., Tsoi P.S. (2025). Citrullination of TDP-43 Is a Key Post-Translation Modification Associated with Structural and Functional Changes and Progressive Pathology in TDP-43 Mouse Models and Human Proteinopathies. bioRxiv.

[139] Hasegawa M., Arai T., Nonaka T., Kametani F., Yoshida M., Hashizume Y., Beach T.G., Buratti E., Baralle F., Morita M. (2008). Phosphorylated TDP-43 in Frontotemporal Lobar Degeneration and Amyotrophic Lateral Sclerosis. Ann. Neurol..

[140] Mosna S., Dormann D. (2025). TDP-43 Phosphorylation: Pathological Modification or Protective Factor Antagonizing TDP-43 Aggregation in Neurodegenerative Diseases?. Bioessays.

[141] Guedes Á.C.B., Santin R., Costa A.S.R., Reiter K.C., Hilbig A., Fernandez L.L. (2017). Distinct Phospho-TDP-43 Brain Distribution in Two Cases of FTD, One Associated with ALS. Dement. Neuropsychol..

[142] Forman M.S., Trojanowski J.Q., Lee V.M.-Y. (2007). TDP-43: A Novel Neurodegenerative Proteinopathy. Curr. Opin. Neurobiol..

[143] Barmada S.J., Serio A., Arjun A., Bilican B., Daub A., Ando D.M., Tsvetkov A., Pleiss M., Li X., Peisach D. (2014). Autophagy Induction Enhances TDP43 Turnover and Survival in Neuronal ALS Models. Nat. Chem. Biol..

[144] Pesiridis G.S., Tripathy K., Tanik S., Trojanowski J.Q., Lee V.M.-Y. (2011). A “Two-Hit” Hypothesis for Inclusion Formation by Carboxyl-Terminal Fragments of TDP-43 Protein Linked to RNA Depletion and Impaired Microtubule-Dependent Transport. J. Biol. Chem..

[145] Watanabe S., Kaneko K., Yamanaka K. (2013). Accelerated Disease Onset with Stabilized Familial Amyotrophic Lateral Sclerosis (ALS)-Linked Mutant TDP-43 Proteins. J. Biol. Chem..

[146] Borgert L., Mishra S., den Brave F. (2022). Quality Control of Cytoplasmic Proteins inside the Nucleus. Comput. Struct. Biotechnol. J..

[147] Lee E.B., Lee V.M.-Y., Trojanowski J.Q. (2011). Gains or Losses: Molecular Mechanisms of TDP43-Mediated Neurodegeneration. Nat. Rev. Neurosci..

[148] Austin J.A., Wright G.S.A., Watanabe S., Grossmann J.G., Antonyuk S.V., Yamanaka K., Hasnain S.S. (2014). Disease Causing Mutants of TDP-43 Nucleic Acid Binding Domains Are Resistant to Aggregation and Have Increased Stability and Half-Life. Proc. Natl. Acad. Sci. USA.

[149] Ceron-Codorniu M., Torres P., Fernàndez-Bernal A., Rico-Rios S., Serrano J.C., Miralles M.P., Beltran M., Garcera A., Soler R.M., Pamplona R. (2024). TDP-43 Dysfunction Leads to Bioenergetic Failure and Lipid Metabolic Rewiring in Human Cells. Redox Biol..

[150] French R.L., Grese Z.R., Aligireddy H., Dhavale D.D., Reeb A.N., Kedia N., Kotzbauer P.T., Bieschke J., Ayala Y.M. (2019). Detection of TAR DNA-binding protein 43 (TDP-43) oligomers as initial intermediate species during aggregate formation. J. Biol. Chem..

[151] Furukawa Y., Kaneko K., Watanabe S., Yamanaka K., Nukina N. (2011). A Seeding Reaction Recapitulates Intracellular Formation of Sarkosyl-Insoluble Transactivation Response Element (TAR) DNA-Binding Protein-43 Inclusions. J. Biol. Chem..

[152] Audrain M., Egesipe A.-L., Tentillier N., Font L., Ratnam M., Mottier L., Clavel M., Le Roux-Bourdieu M., Fenyi A., Ollier R. (2023). Targeting Amyotrophic Lateral Sclerosis by Neutralizing Seeding-Competent TDP-43 in CSF. Brain Commun..

[153] Dhakal S., Wyant C.E., George H.E., Morgan S.E., Rangachari V. (2021). Prion-like C-Terminal Domain of TDP-43 and α-Synuclein Interact Synergistically to Generate Neurotoxic Hybrid Fibrils. J. Mol. Biol..

[154] Loganathan S., Lehmkuhl E.M., Eck R.J., Zarnescu D.C. (2019). To Be or Not to Be…toxic-Is RNA Association with TDP-43 Complexes Deleterious or Protective in Neurodegeneration?. Front. Mol. Biosci..

[155] Yerbury J.J., Farrawell N.E., McAlary L. (2020). Proteome Homeostasis Dysfunction: A Unifying Principle in ALS Pathogenesis. Trends Neurosci..

[156] Rhine K., Li R., Kopalle H.M., Rothamel K., Ge X., Epstein E., Mizrahi O., Madrigal A.A., Her H.-L., Gomberg T.A. (2025). Neuronal Aging Causes Mislocalization of Splicing Proteins and Unchecked Cellular Stress. Nat. Neurosci..

[157] Erdi-Krausz G., Shaw P.J. (2025). Antisense Oligonucleotide Therapy in Amyotrophic Lateral Sclerosis. Curr. Opin. Neurol..

[158] Tseng Y.-L., Lu P.-C., Lee C.-C., He R.-Y., Huang Y.-A., Tseng Y.-C., Cheng T.-J.R., Huang J.J.-T., Fang J.-M. (2023). Degradation of Neurodegenerative Disease-Associated TDP-43 Aggregates and Oligomers via a Proteolysis-Targeting Chimera. J. Biomed. Sci..

[159] Lu S., Hu J., Arogundade O.A., Goginashvili A., Vazquez-Sanchez S., Diedrich J.K., Gu J., Blum J., Oung S., Ye Q. (2022). Heat-Shock Chaperone HSPB1 Regulates Cytoplasmic TDP-43 Phase Separation and Liquid-to-Gel Transition. Nat. Cell Biol..

[160] Park H., Kang J.-H., Lee S. (2020). Autophagy in Neurodegenerative Diseases: A Hunter for Aggregates. Int. J. Mol. Sci..

